# Assessment of leachables in hospital pharmacy compounded topotecan conditioned in common off-label syringes for intravitreal use

**DOI:** 10.1038/s41598-025-24557-9

**Published:** 2025-11-19

**Authors:** William Bello, Camille Hosotte, Camille Stampfli, Antoine Pierrot, Francis L. Munier, Markoulina Berger-Gryllaki, Laurent Carrez, Julian Pezzatti, Farshid Sadeghipour

**Affiliations:** 1 Pharmacy Department, Lausanne University Hospital, Lausanne, Switzerland; 2 School of Pharmaceutical Sciences, University of Geneva, CMU-Rue Michel Servet 1, 1211 Geneva 4, Switzerland; 3 Institute of Pharmaceutical Sciences of Western Switzerland, University of Geneva, University of Lausanne, Lausanne, Switzerland; 4 Center for Research and Innovation in Clinical Pharmaceutical Sciences, Lausanne University Hospital and University of Lausanne, Lausanne, Switzerland; 5 Jules-Gonin Eye Hospital, Fondation Asile des Aveugles, University of Lausanne, Lausanne, Switzerland

**Keywords:** Hospital pharmacy compounding, Prefilled syringes, Topotecan, Intravitreal administration, UHPLC-HRMS, Leachables, Lubricants, Materials for devices, Techniques and instrumentation, Endocrinology, Health care, Risk factors, Materials science, Chemistry, Analytical chemistry, Chemical safety, Environmental chemistry, Polymer chemistry

## Abstract

Compounding drug products is a vital hospital pharmacy feature that requires safe and compatible primary packaging, particularly for prefilled syringes (PFSs). The off-label use of plastic syringes in batch compounding for intravitreal administration has raised concerns over leachables and silicone oil lubricants, which may migrate into drug solutions and potentially cause toxicity. This study evaluated two widely used off-label plastic syringes, BD Plastipak and BBraun Omnifix, for conditioning with topotecan (TPT), a chemotherapeutic used to treat infant retinoblastoma. Leachables were monitored over 12 months via an original LC‒MS method with postcolumn infusion (PCI), including possible detection of silicone degradants. This approach may serve as an indirect method to assess silicone-related contamination due to the spread of free silicone particulates. Both syringes released low overall levels of leachables. However, BD Plastipak emits five oligomeric degradants, two exceeding permitted daily exposure (PDE) limits. In contrast, BBraun Omnifix showed greater chemical stability, releasing only minimal silicone-related compounds and oleamide, suggesting a lower risk for intraocular use. Although these findings support BBraun Omnifix as a safer option, the findings underscore the need for toxicologically assessed, purpose-designed plastic PFSs made from low-extractable materials such as cyclic olefin polymer (COP) or copolymer (COC) for long-term drug storage.

**Supplementary Information:**

The online version contains supplementary material available at 10.1038/s41598-025-24557-9.

## Introduction

Retinoblastoma is the most common intraocular malignancy in children and can be fatal if left untreated^1–3^. The primary goal of retinoblastoma treatment is to prevent metastatic spread and preserve life^2,4^. Chemotherapy-based conservative approaches aim to avoid enucleation and maintain visual function. Intravitreal injection allows direct delivery of high-concentration chemotherapy into the avascular vitreous, bypassing the blood‒retinal barrier and minimizing systemic toxicity through reduced plasma exposure^5,6^.

Topotecan (TPT) has recently emerged as an effective drug for the treatment of intravitreal retinoblastoma, either as monotherapy or in combination with melphalan. It has demonstrated favourable pharmacokinetics, with prolonged vitreous exposure and lower ocular and systemic toxicity in both animal and human studies^5,7,8^. Compared with melphalan alone, combining intravitreal melphalan with TPT improved the control of vitreous seeds and significantly reduced enucleation rates^8,9^.

In this context, TPT was batch-compounded from Hycamtin into multiple microdoses tailored for infant use^10^. Hospital pharmacy compounding is critical when commercial formulations do not meet patient-specific needs. This ensures sterility, dosing precision through gravimetric methods, timely availability and security through quality control. However, in practice, compounding often occurs with limited regulation, funding, and oversight, leading to the off-label use of plastic syringes—raising safety concerns^11,12^.

Syringes used for long-term drug storage may release potentially harmful leachables because of extended contact with formulations, particularly in the absence of risk assessment. Adverse effects have been reported via scientific articles issued from free migrating silicone oil droplets released during injections, including ocular complications such as floaters^13–16^. Despite these risks, no formal requirements exist to evaluate compatibility between drug solutions and primary packaging materials or incentives to monitor leachables and evaluate container-formulation compatibility^11,17,18^. This is particularly concerning for frail and vulnerable populations such as infants, who may be more susceptible to such contaminants^19^.

Lubricants in syringes, used to facilitate movement between the plunger and barrel, are typically silicone-based polymers (e.g., PDMS) or fatty acid derivatives such as oleamide^20,21^. The latter of these substances are often incorporated into syringe polymers during manufacturing and can remain in trace amounts. Their intentional use alongside silicone oils can enhance syringe performance^21–24^. Therefore, it is necessary to assess their migration in drug compounding since they may pose contamination risks.

Given the potential for plastic-related compounds to leach into drug products and that there is no requirement in container compatibility assessment in hospital compounding practices, assessing these substances is vital. Compounds such as BPA and phthalates are known to be present and have endocrine-disrupting effects, posing potential health risks, including reproductive and developmental disorders, in infants^11,17,18^.

Due to the frequent off-label use of plastic syringes in compounding, this paper aims to raise awareness among hospital pharmacists regarding associated risks by studying common containers often selected by hospital pharmacies worldwide. This paper presents a case study evaluating leachables, including lubricants, in TPT prefilled syringes (PFSs), followed by a safety risk assessment via a novel LC‒MS method including postcolumn infusion for hospital pharmacies, which could be a potential means to evaluate leachables and silicone-related compounds, an area often overlooked in conventional leachable testing. This method could serve as a practical marker for hospital pharmacies to evaluate syringe suitability in intravitreal drug delivery as a complementary measure to microscopy. This work aims to inform optimal syringe selection for intravitreal batch compounding and support safer practices in paediatric oncology treatment.

## Experimental section

### Reagents and materials

4,4’-sulfanediylbis[5-methyl-2-(2-methyl-2-propanyl)phenol], 4,4’-(1,3-phenylenedi- 2,2-propanediyl)diphenol, bis(2-ethylhexyl)phthalate-3,4,5,6-d4, 2,4-di-tert-butyl-6-(5-chlorobenzotriazol-2-yl)phenol and bisphenol A-d16 were procured as internal standard at Sigma Aldrich (Gygli, Switzerland). MS-grade water and methanol (MeOH) were purchased from Biosolve (Dieuze, France). LC grade ammonium hydroxide (NH_4_OH) 25% was purchased from Merck (Gygli, Switzerland). Since this experiment is dealing with leachable compounds, liquid solvents were obtained in glass containers to avoid plastic additive contamination. All chromatographic and mass spectral data of standards used in the identification and semiquantitation of compounds, including the limit of detection (LOD) and quantification (LOQ) are found in Tables S1 and S2.

BD Plastipak (Drogheda, Ireland) and BBraun Omnifix-F 1 (Melsungen, Germany), both at 1 mL, were purchased as common syringes used to condition intravitreal PFS and therefore were used to perform the monitoring of leachable compounds, including lubricant-related compounds.

### Leachable study

The time points were selected based on real-world hospital storage scenarios: 4-hour storage simulates immediate post-preparation use, 72-hour storage obeys the recommendation of the manufacturer, 1-month (1 M), 6-month (6 M) and 12-month (12 M) storages support timely availability of inventory stockpiling for urgent surgery and chemotherapy treatment. It is important to note that these time points were also selected according to ICH Q1R2 - testing frequency that should be conducted with minimum 4 time points for an intermediate storage of 12 months^25^.

#### LC-HRMS method

A Thermo Scientific™ ultra-high performance liquid chromatograph Vanquish™ Horizon™ was hyphenated to a Orbitrap™ Q Exactive™ mass spectrometer (Thermo Scientific™, MA, USA), with a heated electrospray ionization (HESI-II) source^17,18,26^.

Analyses were performed on a Waters™ Acquity™ BEH Phenyl (100 × 2.1 mm, 1.7 μm) (Waters™, Milford, MA, USA) and the corresponding VanGuard pre-column. Flow rate and column temperature were set at 0.2 mL/min and 60 °C, respectively. Solvent A (pure water) and solvent B (pure MeOH) were used as mobile phases. The gradient profile used was as follows: a linear ascend from 70% B to 85% B in 6 min, followed by an increase to 95% B in 4 min. An increase to 100% B in 2 min was set, holding at 100% B for 4 min, before returning back at 70% B in 0.1 min and re-equilibrating the column for 9 min. Samples were kept at 10 °C during analyses and a volume of 10 µL was injected.

As for the HESI-II parameters, sheath gas flow rate and auxiliary gas flow rate were set at 30 and 5 arbitrary units, respectively. Capillary temperature at 275 °C and auxiliary heater temperature at 290 °C. Analytes were analysed in positive ion spray voltage at 3 kV. The acquisition program used is a parallel-reaction monitoring (PRM), at a mass resolution of 17’500, at an AGC target of 2 × 10^5^, using a maximum filling time of the C-trap of 50 ms. A normalized collision energy was set at 10%. All chromatograms were acquired using a m/z tolerance of 5 ppm. An isolation window of 1 m/z was set without an isolation offset and multiplexing count. A mass calibration was performed once a week in positive polarity using the Pierce™ Velos ESI Ion Calibration standard mixture (Thermo Scientific™, MA, USA). For Positive ion calibration, the mix consists of n-butylamine, caffeine, MRFA (peptide of Met-Arg-Ala acetate salt) and Ultramark 1621. MS Tune 2.8 (Thermo Scientific™, MA, USA) was used to control the instrument and Chromeleon™ 7.2.7 (Thermo Scientific™, MA, USA) was used to acquire data.

#### Post-column infusion (PCI)

A Chemyx Fusion 100T syringe pump (TX, USA) was used, along with a 10 mL of glass syringe (Hamilton, Nevada, USA) containing 2% ammonium hydroxide in methanol infused at a flow rate of 2 µL per minute.

#### Preparation of prefilled syringes

The preparation of TPT PFS was carried out inside a GMP Class A isolator, located within a GMP Class C cleanroom specifically meant for chemotherapeutic production. This isolator is monitored weekly as per GMP standards to confirm its microbiological quality^10^.

#### Sample preparation

To prepare the samples for the analysis of plastic additives as well as lubricants, the entire 1 mL volume from each syringe was transferred into a vial for analysis. Samples preparations were performed in triplicates of PFS as well as in instrumental injection. Negative controls (syringes without drug) and positive controls (spiked leachables) were also included.

#### Internal standard solution preparation

Stock solution of internal standards (IS) containing five different compounds: bis(2-ethylhexyl)phthalate-3,4,5,6-d4 or DEHP-d4 (IS1), 4,4’-(1,3-phenylenedi-2,2-propanediyl)diphenol or Bisphenol M (IS2), 4,4’-sulfanediylbis[5-methyl-2-(2-methyl-2-propanyl)phenol] or Irganox 415 (IS3), 4-di-tert-butyl-6-(5-chlorobenzotriazol-2-yl)phenol or Tinuvin 357 (IS4) and and bisphenol A-d16 (IS5) was prepared at 100 µg/mL in MeOH. The solution was then diluted 100x with H_2_O/MeOH (1:1) to reach a concentration of 1 µg/mL (work solution).

A blank solution was prepared by spiking a solution of 1 mL containing 0.9% of sodium chloride with 100 µL of the work solution. Samples were also spiked the same way as the blank, before injecting them for analysis.

The IS were attributed to different categories of leachables. IS1 was used to semiquantify plasticisers and silicone-based lubricants, IS2 to semiquantify bisphenol derivatives, IS3 to semiquantify antioxidants, IS4 to semiquantify UV absorbers and stabilizers and IS5 to semiquantify a miscellaneous group of additives including oligomers, non-silicone-based lubricants and others^17,18^.

All internal standards were considered for both semiquantitative approach and IS1 only for estimated quantitative approach. The LOD and LOQ of all internal standards are expressed respectively: IS1 (0.244 and 0.732 ng/mL), IS2 (0.244 and 0.732 ng/mL), IS3 (0.122 and 0.366 ng/mL), IS4 (0.244 and 0.732 ng/mL) and IS5 (0.244 and 0.732 ng/mL)^17,18^.

### Semiquantification approach

#### Evaluation of leachable compounds

The concentrations of Leachables were estimated with the help of the responses factor, calculated according to Eq. (1). The analytes’ concentration was assessed according to Eq. (2). The method is an estimative approach which gives results in the form of estimation of the concentration between 50% and 150% of the true value^17,18,26,27^.

Equation (1) formula used to calculate the relative response factor (RRF) of the internal standards, which consists of considering the response factor of the analyte (RF_compound_) and the RF of the internal standard (RF_IS_).

$${\text{RRFcompound~=}}\frac{{{\text{RFcompound}}}}{{{\text{RFIS}}}}$$

Equation (2) formula used to calculate the concentration of the analyte considering the response of sample (R_sample_) in the form of the area under the curve (AUC) of the analyte and the internal standards, the RRF of the identified analyte and the RF of the internal standards.

$${\text{Csample~=}}\frac{{{\text{Rsample}}}}{{{\text{RRFcompound}}}} \times \frac{{{\text{CIS}}}}{{{\text{RIS}}}}$$

The identification and estimated concentrations were determined using an internal database containing non-silicone-based compounds. The list is found in Table S1-4 in supplementary materials.

#### Study of only lubricant-related compounds

The approach used here is a simple quantification with the use of 1 IS, e.g., DEHP-d_4_ in positive mode. Below are the formulas used to calculate the responses factor according to Eq. (3) and the concentration of the analyte according to Eq. (4)^27^. This method is an approach more estimative than the former which gives a broader range of concentrations, e.g., wider than 50% to 150% of the true value^17,18,27^.

Equation (3) formula used to calculate the response factor (RF) of the internal standards, which consists of pondering the response of the IS in the form of AUC and its known concentration.

$${\text{RFIS~=}}\frac{{{\text{RIS}}}}{{{\text{CIS}}}}$$

Equation (4) formula used to calculate the concentration of the analyte by taking into consideration the response of sample in the form of AUC of the analyte and the RF of the internal standards.

$${\text{Csample~=}}\frac{{{\text{Rsample}}}}{{{\text{RFIS}}}}$$

The identification and estimated concentration of silicone-related compounds are found in Table S1-S4 in supplementary materials.

### Risk assessment

Risk assessment was realised on identified compounds that have undergone estimated quantification. This was achieved by comparing the permissible dose exposure (PDE) with the total daily exposure (TDE)^28–32^. The following equations to perform the risk assessment are found below as follows.

Equation (5) formula for permissible dose exposure (PDE).

$${\text{PDE~[mg/day]~=~}}\frac{{{\text{Estimated~NOAEL~[mg/kg~bw/day]}}}}{{{\text{T1~}} \times {\text{~T2~}} \times {\text{~T3~}} \times {\text{~T4~}}}}{\text{~}} \times {\text{~Body~Weight~[kg]}}$$

Equation (6) formula for maximum daily dose (MDD).

$${\text{MDD~[mL/day]~=~Total~Volume~[mL]~}} \times {\text{~Number~of~daily~Administration~[1/day]}}$$

Equation (7) formula for total daily exposure (TDE).

$${\text{TDE~[ng/day]~=~Estimated~concentration~[ng/mL]~}} \times {\text{~MDD~[mL/day]~}}$$

The no observed adverse effect level (NOAEL), the body weight of the patient, and the multiplied uncertainty factors depending on the parameters of the drug products and the patient are required to calculate the PDE threshold. This is then compared with the total daily exposure (TDE). To calculate the latter, one would require the maximum daily dose (MDD) of the drug product administered to the patient. The estimated concentration of the analyte must be multiplied with the MDD to finally obtain the TDE.

The uncertainty factors (T) are a multiplied series of factors to compensate for series of errors.

Below are so called series of factors elaborated:

- T1 = Interspecies variations.
- T2 = Interindividual variations among humans.
- T3 = from acute to chronic human exposure.
- T4 = from oral to parenteral route.

The NOAEL value is estimated via the calculation of the LD_50_ which is obtained through the EPA T.E.S.T database based on in-silico predictions (extrapolations)^32^. Although it may not be an accurate approach, it is still a quick approach to understand the toxicology of the identified compounds.

## Results and discussion

### Reasoning behind the evaluation of leachable compounds

#### Off-label use of plastic syringes in batch compounding

In a hospital setting, the selection of primary packaging for batch production, such as syringes, infusion bags, vials, and elastomeric pumps, is important when patient care is involved. In this case, syringes are used to contain compounded intravitreal chemotherapeutic drug products such as TPT. In addition to specific reports of excess silicone oil in the medical field, there has been an ongoing trend of off-label use of plastic primary packaging in hospital pharmacy batch compounding^10,33–39^. Immediate to short-term use of plastic syringes is being used in the production of long-term, ready-to-administer PFS for storage. This could represent toxicological issues because these syringes have been accounted for only as immediate-use medical devices based on risk assessments performed by the manufacturer, which makes them safe to use as labelled on the container’s technical sheets. Manufacturers such as BD and BBraun have performed assessments to ensure population safety if their containers are used as intended. However, the storage of filled devices beyond their approved use can lead to the release of unmonitored, potentially harmful, leachable compounds^11^.

#### Adoption of medical device regulatory logics in hospital pharmacy compounding

According to the European Union Medical Device Regulation (EU MDR) and the FDA 510(k), immediate- to short-term use syringes are considered Class 1 syringes because of their low-risk functions; these syringes are used directly in medical wards for general purpose aspirations and the withdrawal of fluids such as diluents, drug products, and body fluids. According to these regulations, industries are obliged to use Class 2 and 3 syringes as long-term storage container closure systems (CCSs) based on good practices for safety of the general population^40,41^. These regulations do not serve hospital pharmacy productions and therefore do not oblige hospital pharmacy compounders to do so. However, industry-based regulatory logic is based on solid scientific data; therefore, hospital pharmacy compounders are inclined to follow regulations to ensure optimal patient safety. MDR regulations state that the intended purpose of a container must align with the data and conclusions generated for the container.

In this context, it would be interesting to show potential leachables in syringes used off-label in the form of case studies to increase awareness by using an analytical method built for assessment of leachables by hospital pharmacies. By considering the risk assessments of leachables studies, hospital pharmacists can advise on safe use in terms of toxicology and clinical risks for patients^11,17,18^.

#### Risk-based approach

According to most extractables and leachables (E&L) experts, the typical E&L protocol is long and expensive to perform in industry, involving a full extractable study on the medical device and then a leachable study on the combined container closure system (CCS) (medical device and drug formulation) based on guidelines^40–46^. In response, many industries are proceeding towards simplifying the E&L procedure. If hospital pharmacies are to perform risk assessment of their batch products, they would do best to adopt a similar approach. A risk-based approach in hospital pharmacy leachable assessment should be composed of different variables: route, frequency and duration of administration, type of patients, and formulation. TPT PFS can be considered a medium-risk product because it not only uses an off-label medical device but is also used for parenteral administration to frail and vulnerable patients, such as infants^11^. This approach would also include an estimative quantification with an estimative toxicology. All the analytical procedures and a comprehensive toxicological assessment are expensive, demanding high cost and time, which makes this approach necessary as a means of first-step measurement. Moreover, this estimative approach underestimates the value, making the toxicological threshold value of a given compound in exposure to the patient lower, adding an extra layer of protection for frail and vulnerable patients such as neonates and infants that are physiologically different from those of the general adult population.

### Materials for the construction of syringes used in hospital pharmacy compounding

Both BD Plastipak and BBraun Omnifix 1 mL syringes consist of a barrel made of polypropylene and a plunger head made of isoprene rubber. The barrel has a black graduation scale printed with ink on its surface to enable estimation of volume measurement of aspiration and withdrawal of liquid. The plunger head and the inner surface of the barrel are sprayed with PDMS medical-grade lubricant to facilitate smooth movement of the plunger^20–24^. Additionally, a label is affixed to the barrel to allow product distinction once the syringe is filled with the drug product to become a CCS but does not possess a low extractable profile quality.

### Leachable studies

Three batches of TPT in BD Plastipak and BBraun Omnifix were analysed at six different time points, i.e., initial, 4 h, 72 h, 1 month, 6 months, and 12 months, at a refrigerated temperature, which was deemed the appropriate storage condition for this product on the basis of a conducted stability study^10^. There are two parts to this study: the first is the evaluation of leachables at the product expiration date, which is 12 months, to study the outcome of leachable compounds in these two common syringe types, and the second part is the monitoring of only lubricant-related compounds and their degradants at all time points to study the trend of degradation for these two syringe types.

Tables S1-4 compile the leachable compound data observed in both syringes, including identification methods, retention times, m/z values and adducts, and semiquantitative concentrations as well as toxicological assessment data, are in the supplementary material.

#### Evaluation of leachables

##### Identification of leachables and functional implications

After 12 months of storage, both syringes presented similar patterns of leachable compounds, all of which were observed at low concentrations (between 0.1 and 15 ng/mL). BD Plastipak produced three times more diethylhexyladipate (DEHA) than did BBraun Omnifix. This could be due to the medical device formulation. DEHA is an important plasticizer that is responsible for ensuring flexibility in primary packaging and preventing brittleness^47^. It is fused with the polymer before extrusion through van der Waals interactions. Due to this weak bonding, DEHA compounds are easily removed by aqueous solutions, which explains the observed concentrations. Moreover, two other plasticizers were observed at very low concentrations only in BD Plastipak (sebacate and citrate derivatives).

Degradants of antioxidants were observed in both primary packages (i.e., 2,6-di-tert-butyl-4-hydroxy-4-methylcyclohexa-2,5-dien-1-one, 2,6-di-tert-butyl-p-cresol, 3-(3,5-di-tert-butyl-4-hydroxyphenyl)propanoic acid, 3-(3-5-di-tert-butyl-1-hydroxy-4-oxo-2,5-cyclohexadiene-1-yl)propanoic acid, and 3,5-di-tert-butyl-4-hydroxybenzyl alcohol). All these compounds are derived from 2,6-di-tert-butyl-p-cresol (BHT) and 3-(3,5-di-tert-butyl-4-hydroxyphenyl)propanoic acid (fenozan). BHT may be a small standalone antioxidant or a part of a more complex structure and fenozan is part of a larger structure (i.e., Irganox 1010, 1076, etc.). According to our observations, BD Plastipak released three times more of fenozan than BBraun Omnifix, which could be due to the presence of either more complex antioxidants or the instability of the polymer due to long-term storage. However, BBraun Omnifix syringes released the greatest number antioxidant derivatives. There could be a higher oxygen content in the drug formulation, which induced the oxidation of fenozan and BHT into their oxidated states (hydroxy, aldehyde, ketone), related to the porosity of the material.

Markers of rubber deterioration were also observed in BD Plastipak syringes. These compounds are rubber cross-linkers used in the curing of rubber. Usually, rubber plunger heads are thermoplastic elastomers and therefore should not require rubber crosslinkers, which would be more appropriate for thermoset elastomers. Nevertheless, low concentrations of rubber cross-linkers were observed at 12 months of storage.

Ink graduation markers were observed in both syringes. BBraun Omnifix has shown traces of acrylate derivatives (triethyleneglycol dimethacrylate) and caprolactam at low concentrations, whereas BD Plastipak released twice as much caprolactam than BBraun Omnifix. Moreover, bisphenol A (BPA) and phosphate flame retardants (triphenyl phosphate and tris(2-chloro-1-methylethyl) phosphate) were observed in greater amounts in BD Plastipak than in BBraun Omnifix. BPA was not found in the latter, but this would require more interbatch analysis to confirm its presence since it can appear in one batch and not another. Flame retardants have no place in syringes since their role is to prevent the spread of fire through electric cables and equipment. These compounds, including BPA, could result from industrial in-process contamination^17,18^.

##### Estimative toxicology of leachables

**Fig. 1:**
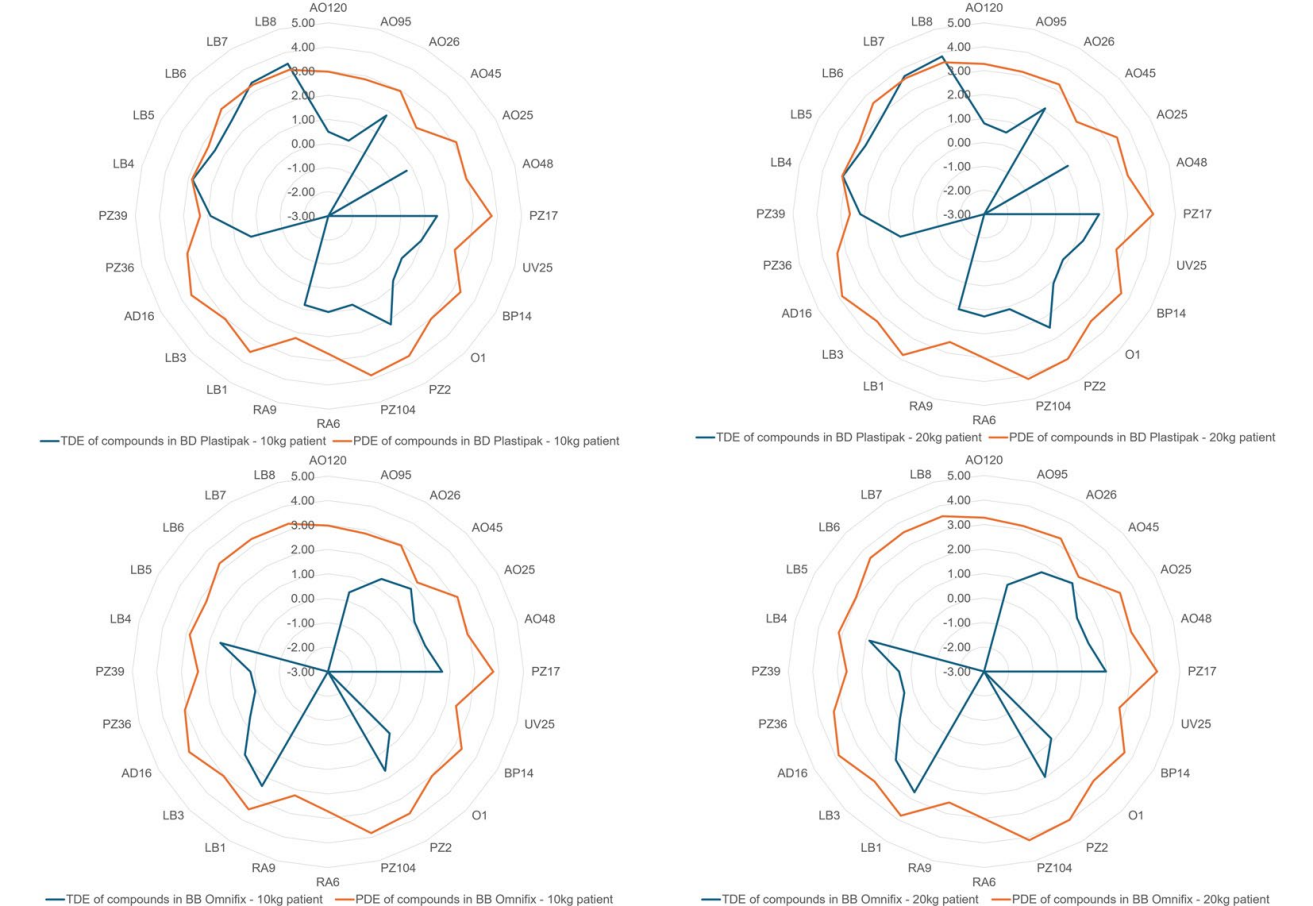
Risk assessment of topotecan PFS stored in BD Plastipak and BB Omnifix primary packaging after twelve months in refrigerated temperature. The compounds (along with the laboratory codes) identified, semiquantified and assessed are: 2,6-di-tert-butyl-4-hydroxy-4-methylcyclohexa-2,5-dien-1-one (AO120), 2,6-di-tert-butyl-p-cresol (AO95), 3-(3,5-di-tert-butyl-4-hydroxyphenyl)propanoic acid (AO26), 3-(3-5-di-tert-butyl-1-hydroxy-4-oxo-2,5-cyclohexadiene-1-yl)propanoic acid (AO45), 3,5-di-tert-butyl-4-hydroxybenzaldehyde (AO25), 3,5-Di-tert-butyl-4-hydroxybenzyl alcohol (AO48), Acetyl tributyl citrate (PZ17), Benzotriazole (UV25), Bisphenol A (BP14), Caprolactam (O1), Di-(2-ethylhexyl) adipate (PZ2), Di-(2-ethylhexyl) sebacate (PZ104), N, N-Dibutylformamide (RA6), N-butylformamide (RA9), Oleamide (LB1), Tetradecanamide (LB3), triethyleneglycol dimethacrylate (AD16), Triphenyl phosphate (PZ36), Tris(2-chloro-1-methylethyl)phosphate (PZ39), hexamethylcyclotrisiloxane (LB4), octamethylcyclotetrasiloxane (LB5), decamethyltetrasiloxane (LB6), decamethylcyclopentasiloxane (LB7), dodecamethylpentasiloxane (LB8).

To estimate the risk of critical patients being exposed to potentially hazardous compounds, it is important to first determine the MDD of TPT, which is calculated at 1 mL, since it is administered only intravitreally or intracamerally during surgery. Next, PDE and TDE were calculated according to Eq. 3 and Eq. 5 for nonlubricant leachables. As a result, none of the TDEs of the identified compounds related to leachables surpassed their PDE thresholds, as shown in Fig. 1. DEHA was nearly the highest in concentration; however, many E&L experts say that this is normal. According to some toxicologists, DEHA can be viewed to be toxic with multiple endpoints, such as neurotoxic, hepatotoxic, and cardiotoxic, and a potential endocrine disruptor compound (EDC) of concern if infant patients are involved in the treatment^48^. More tests are needed to determine the safety of patients when exposed to off-label containers that have been used to store high-risk products.

Chromatograms and mass spectra in positive and negative modes are available in Figs. S1-S13 in the supplementary materials.

#### Monitoring of lubricant-related compounds and their degradants

Six different time points—initial, 4 h, 72 h, 1 month, 6 months, and 12 months—were considered at refrigerated temperature, which was deemed the appropriate storage condition for this product based on the stability study^10^.

As mentioned in section “ Leachable studies”, Tables S1-4 compile the leachable compound data observed in both syringes, including identification methods, retention times, m/z values and adducts, and semiquantitative concentrations, as well as toxicological assessment data, and are in the supplementary material.

The monitoring of lubricants via a UHPLC-HRMS platform is challenging in terms of compound detectability and, to our knowledge, has not been attempted before due to instrument incompatibility. However, with the installation of PCI, as described in section “Post-column infusion (PCI)”, it is possible to identify and estimate the concentrations of some silicone degradants or oligomers. The main objectives of this study were twofold: (1) identify any non-silicone-related lubricants and any silicone-related degradants and (2) evaluate the toxicological risk of these compounds after long-term storage in patients for intravitreal administration to treat retinoblastoma.

**Fig. 2:**
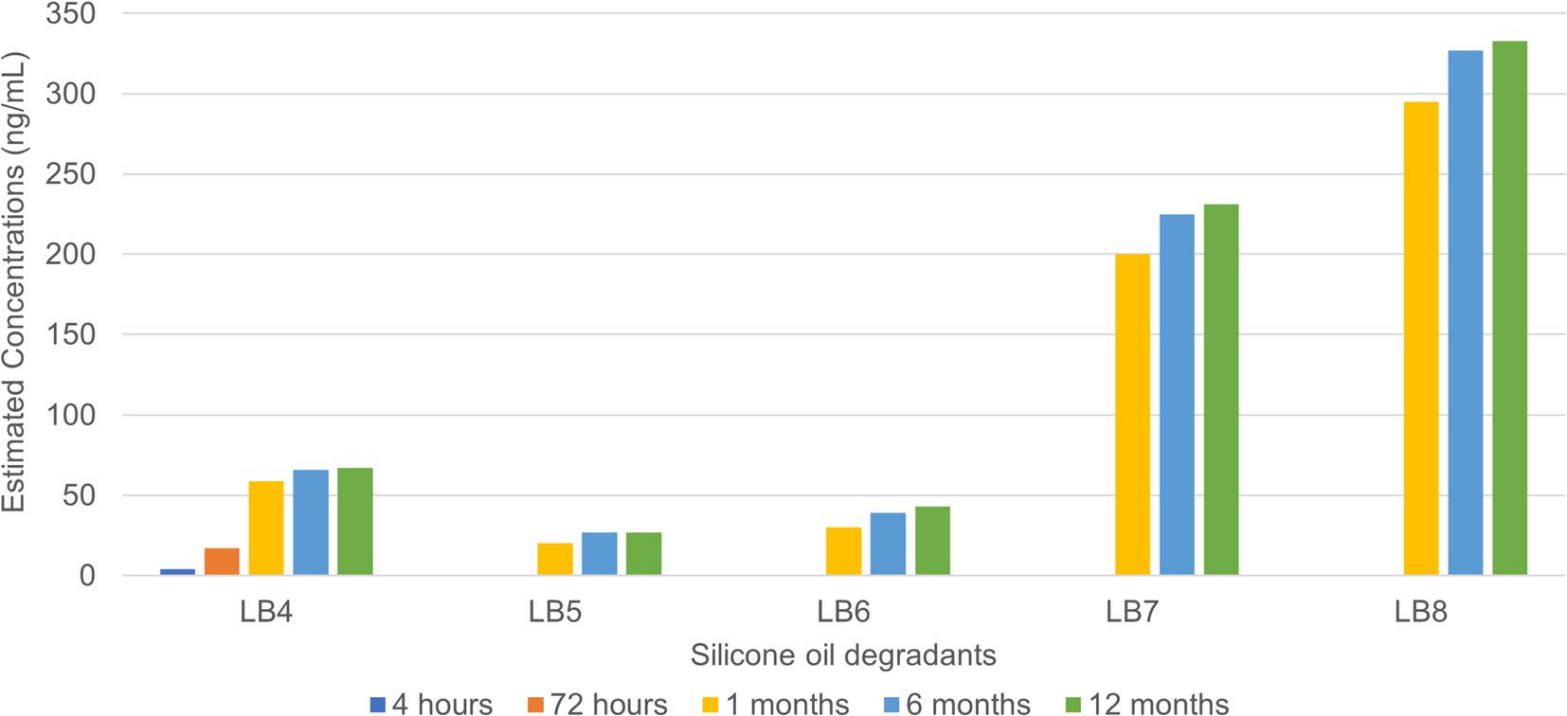
The release of silicone-based degradant/oligomers evolving in terms of estimated concentrations at different time points over a period of 12 months in the BD Plastipak. The compounds (along with the laboratory codes) identified, semiquantified and assessed are: hexamethylcyclotrisiloxane (LB4), octamethylcyclotetrasiloxane (LB5), decamethyltetrasiloxane (LB6), decamethylcyclopentasiloxane (LB7), dodecamethylpentasiloxane (LB8).

##### Identification of lubricant-related compounds

As illustrated in Fig. 2, five different silicone-related degradants were identified in the BD Plastipak syringe, and their concentrations were estimated to range from 4 to 300 ng/mL after 12 months of material‒solution contact. The concentration of these oligomers tends to increase slowly over time because of their instability in aqueous solution, especially when the device is not meant to be used as a storage device for more than 72 h, reflecting the quality of the silicone oil lubricant. From the initial time point to 72 h, little to no detectable presence of silicone-based oligomers was observed within the sensitivity limits of our analytical platform. The concentrations of these silicone-based oligomers are potentially too low to cause toxicological issues for patients of all ages, including infants and neonates for short-term use of these syringes for up to 72 h, which are labelled and strongly advised by the manufacturer, for all intents and purposes. However, as these syringes are used outside their regulated boundaries, an increase in the concentration of these oligomers is observed after 6 months, which is to be expected, especially with LB7 and LB8 having higher estimated concentrations than the rest. The increase in the concentration of all oligomers could be related to a small volume effect. Interestingly, at 6 months, an exhaustive asymptotic curve is observed, forming a plateau when the syringes are left in storage for 12 months. The stagnation appears after 6 months for all oligomeric silicone degradants, which is due to the equilibrium between the PDMS polymer and the oligomeric degradants. This reflects the solubility of the oligomers of interest and the tendency of PDMS to break down into oligomers due to steric hindrances and other molecular stresses. This depends on the angle of attack, which results in the formation of cyclic and linear silicone degradants. There was a four- to seventeen-fold increase between the initial filling in primary packaging and after twelve months of storage.

To understand slightly more about silicone oil, or PDMS, it is a popular lubricant used to enable frictionless surface transition between two objects. PDMS is a low-density polymer composed of silanol monomers. However, since they are linked by silanol bonds, they could be prone to hydrolysis when in contact with aqueous solution. When these bonds break oligomers, either linear or cyclic structures are formed and tend to leach into the drug solution^49^.

The identified degradants are siloxane and silane in nature and were detected in ESI positive mode as [M + H]^+^ adduct. The identification of silicone derivatives via UHPLC-HRMS is particularly unusual because these compounds are volatile to semivolatile by nature. However, the design of the current instrument layout enabled the identification of some siloxanes and silanes due to the installation of a PCI using ammonium hydroxide (NH_4_OH) at very low concentrations and at low flow rates^17,18^. It is hypothesized that the PCI transforms the ion source by creating an environment rich in proton and ammonium molecules, forcing the creation of adducts of silanol and siloxanes substances.

With respect to possible container‒content interactions, describing any interaction with a TPT drug formulation is difficult. It is not known if leachables interact with topotecan and its excipients until we observe signs of physical‒chemical phenomena, which are related to incompatibility between the drug formulation and the medical device. All we can do is describe the presence and concentration of leachables in the solution that may end up in the patient’s bloodstream via means of treatment. However, one good observation is that, on the basis of the results of the TPT stability study, TPT concentrations were not significantly affected since we established that they remained stable for 12 months in BBraun Omnifix Syringes and BD Plastipak Syringes.

**Fig. 3:**
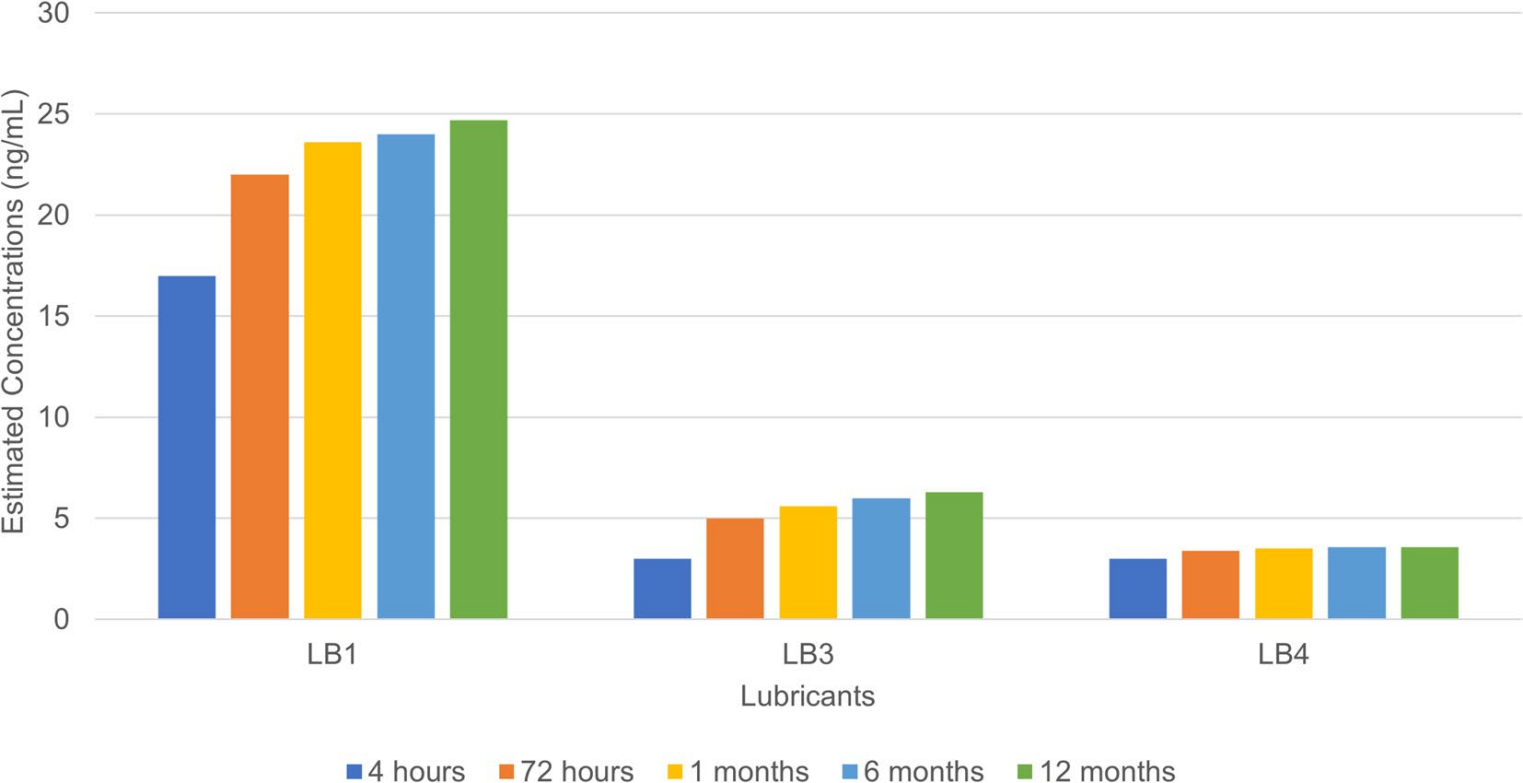
The release of oleamide and its degradants as well as silicone-based oligomeric contaminant evolving in terms of estimated concentrations at different time points over a period of 12 months. The compounds (along with the laboratory codes) identified, semiquantified and assessed are: Oleamide (LB1), Tetradecanamide (LB3), Hexamethylcyclotrisiloxane (LB4).

For BBraun Omnifix, as shown in Fig. 3, small concentrations of oleamide (LB1) were observed after 4 to 72 h of contact with the solution, i.e., between 20 and 25 ng/mL. The oleamide degradant tetradecanamide (LB3) was identified and semiquantified at concentrations less than 5 ng/mL. Oleamide degradation is usually linked to its intrinsic quality and is not related to in situ reactions with aqueous drug solutions. There was no further increase in concentration after 4 h. Oleamide and its degradant are lipophilic by nature^50,51^. This small molecule is an oleic acid derivative with an amide functional group that renders the molecule inert to acidic and basic compounds. Its original purpose was to serve as a slip agent; it is added at a small concentration during the moulding phase of the medical device and remains incorporated into the polymer, which explains its presence in the drug formulation. As an alternative to silicone-based lubricants, oleamide can be a very interesting choice for use in syringes and vials containing aqueous medication. It requires small concentrations to perform optimally as a lubricant^50,51^. Although it was identified at a small concentration, it was never added to the technical sheet because it served no purpose as a lubricant, and it has been assessed by E&L experts as safe before market release. After 12 months of storage, there was a negligible increase in oleamide concentration of approximately twofold. A silicone-related degradant called hexamethylcyclotrisiloxane (LB4), which comes from silicone oil as part of lubrification, was also detected.

The observation of oleamide in BBraun Omnifix syringes is particularly interesting since both syringes are sprayed with free silicon oil on the barrel and the plunger head^52,53^. According to our observations, the spraying technology of free silicone oil in BD Plastipak may differ from that of BBraun Omnifix. The former may have contributed to the added presence of silicone oligomers. It is even possible that there may be less free silicone oil sprayed in BBraun Omnifix, which makes sense when oleamide, which is already incorporated in rubber, is used as a secondary lubricant, hence reducing the reliance on silicone oil.

##### Estimative toxicology of lubricant-related compounds

In terms of risk assessment, as shown in Fig. 1, the only compounds whose TDE surpassed its PDE appeared to be decamethylcyclopentasiloxane (LB7) and dodecamethylpentasiloxane (LB8) at 1 to 12 months of batch storage, which could be considered nonrecommended for long-term contact exposure between this syringe and the drug formulation. Judging by the trend on the histogram shown in Fig. 2, the start of saturation seemed to be visible at the 1-month time point. Unfortunately, to clarify further, it would have been optimal to possess data at the 72-hour time point, but due to the high LODs for these compounds, the concentrations of LB7 and LB8 were not measurable and no models were constructed using their standards to estimate concentrations lower than their LODs. However, it is assumed that their concentration would be much lower than that at the 1-month time point since the lubricant is of medical grade and does not easily degrade when in contact with the formulation for less than 6 days. Importantly, Class 1 syringes are labelled for acute use for simple actions and, in the worst case, for short-term storage for up to 72 h. Therefore, their use for longer than 72 h, from 1 month to 12 months, for example, would not be recommended for infant patients. The other silicone degradants (LB4, LB5 and LB6) were found at relatively low concentrations, which put their TDEs below their PDE after 72 h, up to 12 months. Although syringes seem safe for use in adults, the use of these syringes in infant patients after 72 h of batch storage of medium- to high-risk compounding formulations, such as the current compounding PFS, it strongly discouraged.

In contrast, for BBraun Omnifix, although it may also seem to be a container misappropriation, it did not surpass the TDE-PDE threshold. There has been very little evolution of oleamide (LB1) and its degradant, tetradecanamide (LB3). In terms of safety risk assessment, none of the TDEs surpassed their PDE thresholds, which appears to be safe for use for up to 12 months. The use of only a silicone-based oligomeric degradant was also estimated to be safe because the TDE of the compound did not surpass its PDE threshold. Tables S3 and S4 compile the leachable compound toxicological data observed in both syringes in the supplementary material, including TDE and PDE.

In conclusion, BBraun Omnifix could be a good fit to condition TPT as a PFS, but would it be appropriate for long-term storage? More tests are needed to determine its safety to ensure that no potentially harmful entities can cause issues in frail and vulnerable patients. Moreover, the migration of free silicone oil can be the cause of floaters being injected into patients’ eyes. The appropriate use of primary packaging should be encouraged to reduce incidence, and the promotion of an E&L-related risk assessment should be undertaken to assure patient safety^11,14,15^.

### Endocrine disruptor identification

Ten potential EDCs were identified in BD Plastipak (AO95, PZ17, UV25, BP14, PZ2, PZ36, PZ39, LB4, LB5, and LB7) in comparison to six in BBraun Omnifix (AO95, PZ17, PZ2, PZ36, PZ39, and LB4), nearly half the amount of the former. Although some of these compounds are found at low concentrations, EDCs are particular compounds that do not follow regular toxicology; they can have toxicological effects on the growth and development of younger patients, especially at low concentrations^54^. The compounds that contributed to the increase in EDCs in the BD Plastipak syringes were silicone-based oligomeric degradants. In contrast, BBraun Omnifix did not seem to release many EDCs because of the low contribution of lubricant-related compounds. All EDC information is compiled in Tables S3 and S4 in the supplementary information.

### Limitations

It is essential to address several key limitations in analytics, toxicology, and comparative studies in this paper as these can significantly impact the reliability and interpretation of research findings.

#### Analytical limitations

First, monitoring volatile, semivolatile, and nonvolatile leachable compounds would be ideal for this type of study because of the level of risk of exposure to frail and/or vulnerable patients, such as infants. The current analytical method uses a fixed list of 200 commonly observed compounds, that are of toxicological concerns; therefore, compounds that are not listed cannot be assessed, which is part of the limitation of the method, and its semiquantitation method allows its user to estimate the compounds that are listed in the database. However, the silicone-related compounds were estimated with a larger margin of error as an estimation. It tends to overestimate the concentration because of the selection of the IS used. Moreover, silicone degradants were identified, and their concentrations were estimated via PCI-integrated LC‒MS. Due to the limitations of the instrument, not all degradants were identified; therefore, analysis via GC‒MS would have been an optimal method for analysing volatile and semivolatile compounds. Furthermore, it is concerning that any presence of oligomers could be related to interbatch variation. Silicone degradation can vary from batch to batch, which is why E&L must be performed. Finally, leachable assessments of other storage conditions (RT and freezing temperature) of the TPT PFS were not conducted. If this is done properly, it would yield multidimensional results, establishing the influence of temperature on the release of leachables and even their stability.

#### Toxicological limitations

A full toxicological assessment would have been adequate for this type of study. However, this approach could demand greater cost and more time to establish, which makes this current approach necessary as a means of first-step measurement.

#### Comparative study limitations

Finally, TPT conditioned in syringes made of COP and COC could have been used as a comparison to the other two syringes to demonstrate the low-extractable profile marketed and recommended for long-term storage of DPs^17,18^. However, there was also an intention to demonstrate case studies of off-label use of primary packaging. A glass syringe would have been the optimal control sample. While glass syringes are more inert, their use in ophthalmic surgery and other interventions is contraindicated because of the risk of ocular trauma from glass particles and plunger malfunction. There are many inconveniences due to the high degree of breakage and risk of particulates.

### Expert opinions

#### The use of appropriate primary packaging material

The off-label use of plastic primary packaging, including syringes, should not be underestimated. Both syringes tested herein, and many others that are not mentioned in this paper have already been assessed by manufacturers to be safe for use as intended as per their label, which is for simple aspiration and withdrawal of fluids, whether it is a drug formulation, a diluent, or even a biological fluid. Therefore, these items should not be used outside of their approved use for safety reasons. Industrial drug products intended for long-term batch production are conditioned in primary packaging materials that have been previously risk assessed by industrial E&L experts to ensure optimal patient safety for product market access. Therefore, as an expert opinion, hospital pharmacy compounders should consider doing the same for future endeavours. A recommendation to hospital pharmacy compounders would be the use of Class 2 and 3 syringes made of COP or COC, which are better suited for all drug formulations, including sensitive drug delivery, such as biologics and ophthalmic drug formulations, featuring low extractables and low silicone particle burden^40,41,55,56^. Melo et al. reported that there is often a lack of intraocular primary packaging available, forcing medical institutions to use syringes with free silicone oil. COC- and COP-made syringes could be optimal candidates and result in smaller volumes for low-volume administrations^14,15,55–57^.

#### Promotion of leachable assessment

Another point to recommend would be leachable risk assessment in hospital pharmacy batch compounding. Any off-label use of Class 1 syringes could be safe for adults, but probably not for infants or neonates. As an expert opinion, it would be ideal to perform a risk-based approach on medium- and high-risk drug products. The degree of risk should be based on the route of administration, the type of patients, the type of formulation, the selected medical devices, the frequency of administration, and the duration of treatment. This could be set up in the form of a workflow that could be adaptable, standardizable, affordable, and applicable for hospital pharmacy compounding practices^11^.

If off-label primary packaging is used because the appropriate packaging is deemed too costly to implement in hospital pharmacy production, it is good practice to at least perform an extractable study to ensure that batch-produced products are safe for use in frail and vulnerable patients. Although no regulation exists that obliges compounders to assess the E&L-related risk of PFS, compounders are ethically and morally liable to ensure a safe product for patients.

If appropriate packaging, such as a COC or COP syringe, is implemented in a hospital pharmacy batch compounding to bring about a PFS product to patients but it is too costly to perform a complete risk assessment, compounders can request a technical report issued from E&L studies from the manufacturer. The results can be used as a basis for the compounder’s product to estimate the safety of the product. In terms of extra safety measures, an extractable study can always be performed on the medical device, which does not cost as much as a full assessment.

## Conclusion

In conclusion, given the widespread off-label use of plastic syringes in hospital compounding, particularly for paediatric oncology and ocular therapies, this work aims to raise awareness about potential toxicological risks from packaging-derived contaminants. Both commonly used syringes showed similar leachable profiles (up to 12 months), remaining within PDE safety thresholds under this analytical approach. However, concerning lubricant-related analysis, BD Plastipak syringes exhibited two silicone-based oligomeric degradants after long-term storage that exceeded toxicological limits, indicating that they may be unsuitable for extended storage or intraocular use beyond 72 h. In contrast, BBraun Omnifix syringes released only non-silicone-based lubricants (oleamide and tetradecanamide) in safe amounts, possibly due to lower internal free silicone oil levels.

This study also introduces a innovative UHPLC-HRMS method with postcolumn infusion to assess leachable compounds, including silicone-based lubricants, in two commonly used off-label syringes (BD Plastipak and BBraun Omnifix) filled with TPT. The method successfully identified silicone-related degradants, offering a practical marker for hospital pharmacies to evaluate and estimate syringe suitability in intravitreal drug delivery—an area often overlooked in conventional leachable testing.

These findings underscore the importance of evaluating primary packaging in sterile compounding. BBraun Omnifix syringes may offer a safer option for intravitreal TPT preparation, although comprehensive leachable profiling, including GC‒MS for volatiles and complementary techniques, is essential to perform a full assessment before routine clinical adoption.

Ultimately, this study emphasizes the need for proactive risk assessment and stricter regulatory guidance to ensure the safety of vulnerable patients receiving high-risk drug compounding.

## Supplementary Information

Below is the link to the electronic supplementary material.

Supplementary Material 1

## Data Availability

Data is provided within the manuscript or supplementary information files.

## References

[1] Ortiz, M. V. & Dunkel, I. J. Retinoblastoma. *J. Child. Neurol.* **31** (2), 227–236. 10.1177/0883073815587943 (2016). 10.1177/0883073815587943 26023180

[2] Munier, F. L. et al. Conservative management of retinoblastoma: challenging orthodoxy without compromising the state of metastatic grace. Alive, with good vision and no comorbidity. *Prog. Retin Eye Res.* **73**, 2563. 10.1016/j.preteyeres.2019.05.005 (2019). 10.1016/j.preteyeres.2019.05.005 31173880

[3] Ancona-Lezama, D., Dalvin, L. A. & Shields, C. L. Modern treatment of retinoblastoma: a 2020 review. *Indian J. Ophthalmol.* **68** (11), 2356–2365. 10.4103/ijo.IJO_721_20 (2020). 33120616 10.4103/ijo.IJO_721_20 PMC7774148

[4] Bossacoma, F. et al. Optimizing the storage of chemotherapeutics for ophthalmic oncology: stability of Topotecan solution for intravitreal injection. *Ophthalmic Genet.* **41** (4), 397–400. 10.1080/13816810.2020.1776336 (2020). 32490703 10.1080/13816810.2020.1776336

[5] Del Sole, M. J. et al. Ocular and systemic toxicity of high-dose intravitreal Topotecan in rabbits: implications for retinoblastoma treatment. *Exp. Eye Res.* **218**, 109026. 10.1016/j.exer.2022.109026 (2022). 35276184 10.1016/j.exer.2022.109026 PMC9502017

[6] Buitrago, E. et al. Ocular and systemic toxicity of intravitreal Topotecan in rabbits for potential treatment of retinoblastoma. *Exp. Eye Res.* **108**, 103–109. 10.1016/j.exer.2013.01.002 (2013). 23333535 10.1016/j.exer.2013.01.002

[7] Nadelmann, J. F., Brodie, J. H., Muca, S. E. & Abramson, E. Is intravitreal Topotecan toxic to retinal function? *Br. J. Ophthalmol.* **105** (7), 1016–1018. 10.1136/bjophthalmol-2020-316588 (2021). 32665221 10.1136/bjophthalmol-2020-316588 PMC9446382

[8] Kiratli, H., Koc, I., Ozturk, E., Varan, A. & Akyuz, C. Comparison of intravitreal Melphalan with and without Topotecan in the management of vitreous disease in retinoblastoma. *Jpn J. Ophthalmol.* **64** (4), 351–358. 10.1007/s10384-020-00743-2 (2020). 32447585 10.1007/s10384-020-00743-2

[9] Yousef, Y. A. et al. Safety and efficacy of intravitreal chemotherapy (Melphalan) to treat vitreous seeds in retinoblastoma. *Front. Pharmacol.* **12**, 696787. 10.3389/fphar.2021.696787 (2021). 34322023 10.3389/fphar.2021.696787 PMC8311556

[10] Bello, W. et al. Stability study of Topotecan in ophthalmic prefilled syringes for intravitreal delivery. *Eur. J. Hosp. Pharm. 2025 Jan* **20**, ejhpharm–2024. 10.1136/ejhpharm-2024-004346 (2024). 10.1136/ejhpharm-2024-004346 PMC12772580 39837598

[11] Bello, W. et al. A critical opinion-based review of hospital pharmacy compounding with respect to the risk of leachable substances due to the off-label use of plastic primary packaging. *Ther. Adv. Drug Saf.* **16**, 20420986251317424. 10.1177/20420986251317424 (2025). 39958972 10.1177/20420986251317424 PMC11826846

[12] PIC/S Guide to Good Practices for the Preparation of Medicinal Products in Healthcare Establishments (PE 010–4). Pharmaceutical Inspection Co–operation Scheme (2014).

[13] Mule, D. et al. Drug-Leachable interaction product evaluation in prefilled syringe of Ganirelix acetate injection. *J. Pharm. Sci.* **113** (8), 2023–2027. 10.1016/j.xphs.2024.05.012 (2024). 38796153 10.1016/j.xphs.2024.05.012

[14] Melo, G. B., Shoenfeld, Y. & Rodrigues, E. B. The risks behind the widespread use of siliconized syringes in the healthcare practice. *Int. J. Retin Vitr*. **7**, 66. 10.1186/s40942-021-00338-0 (2021). 10.1186/s40942-021-00338-0 PMC8557543 34717776

[15] Melo, G. B. et al. Release of silicone oil droplets from syringes. *Int. J. Retin Vitr*. **5**, 1. 10.1186/s40942-018-0153-8 (2019). 10.1186/s40942-018-0153-8 PMC6318836 30788149

[16] Bijon, J., Mundae, R., Fisher, Y. & Freund, K. B. Multiple small floaters associated with silicone oil droplets following intravitreal Pegcetacoplan injection. *JAMA Ophthalmol.* **141** (9), 907–909. 10.1001/jamaophthalmol.2023.3495 (2023). 37561447 10.1001/jamaophthalmol.2023.3495 PMC10416084

[17] Bello, W., Pezzatti, J., Berger-Gryllaki, M., Rudaz, S. & Sadeghipour, F. Development of a generic approach for monitoring leachable compounds in hospital pharmacy-prepared prefilled plastic packaging by ultrahigh-performance liquid chromatography coupled to high-resolution mass spectrometry with postcolumn infusion. *J. Pharm. Biomed. Anal.* **236**, 115640. 10.1016/j.jpba.2023.115640 (2023). 37683372 10.1016/j.jpba.2023.115640

[18] Bello, W., Pezzatti, J., Rudaz, S. & Sadeghipour, F. Study of leachable compounds in hospital pharmacy-compounded prefilled syringes, infusion bags and vials. *J. Pharm. Sci.* **113** (11), 3227–3237. 10.1016/j.xphs.2024.08.004 (2024). 39173742 10.1016/j.xphs.2024.08.004

[19] International Council for Harmonisation of Technical Requirements for Pharmaceuticals for Human Use. ICH Q3E: Concept paper for the development of a guideline for extractables and leachables (2020). https://database.ich.org/sites/default/files/ICH_Q3E_ConceptPaper_2020_0710.pdf.

[20] Jenke, D. et al. Evaluation of the general solution compatibility of polymer materials used in medical devices such as syringes. *PDA J. Pharm. Sci. Technol.* **66** (4), 286–306. 10.5731/pdajpst.2012.00869 (2012). 10.5731/pdajpst.2012.00869 22767879

[21] Hahladakis, J. N., Velis, C. A., Weber, R., Iacovidou, E. & Purnell, P. An overview of chemical additives present in plastics: migration, release, fate and environmental impact during their use, disposal and recycling. *J. Hazard. Mater.* **344**, 179–199. 10.1016/j.jhazmat.2017.10.014 (2018). 29035713 10.1016/j.jhazmat.2017.10.014

[22] Makwana, S., Basu, B., Makasana, Y. & Dharamsi, A. Prefilled syringes: an innovation in parenteral packaging. *Int. J. Pharm. Investig*. **1** (4), 200–206. 10.4103/2230-973X.93004 (2011). 23071944 10.4103/2230-973X.93004 PMC3465144

[23] Dulal, N. et al. Slip-additive migration, surface morphology, and performance on injection moulded high-density polyethylene closures. *J. Colloid Interface Sci.* **505**, 537–545. 10.1016/j.jcis.2017.06.040 (2017). 28645037 10.1016/j.jcis.2017.06.040

[24] Bishop, C. A.* Vacuum Deposition Onto Webs, Films and Foils* 149 (William Andrew, 2015).

[25] International Council for Harmonisation (ICH). *ICH Harmonised Tripartite Guideline. Stability Testing of New Drug Substances and Products Q1A(R2)* (ICH Secretariat, 2003).

[26] Bello, W., Pezzatti, J., Rudaz, S. & Sadeghipour, F. Development of a generic sample Preparation method using dispersive liquid-liquid Microextraction for the monitoring of leachable compounds in hospital pharmacy-prepared prefilled drug products. *Anal. Methods*. **16** (11), 1697–1707. 10.1039/d3ay02234j (2024). 38421023 10.1039/d3ay02234j

[27] Christiaens, P., Beusen, J. M., Verlinde, P., Baeten, J. & Jenke, D. Identifying and mitigating errors in screening for organic extractables and leachables: part 2-Errors of inexact identification and inaccurate quantitation. *PDA J. Pharm. Sci. Technol.* **74** (1), 108–133. 10.5731/pdajpst.2018.009779 (2020). 10.5731/pdajpst.2018.009779 31308065

[28] Ball, D. J. & Beierschmitt, W. P. Permitted daily exposure values: application considerations in toxicological risk assessments. *Int. J. Toxicol.* **39** (6), 577–585 (2020). 10.1177/1091581820946746 32794434

[29] Jenke, D. & Carlson, T. A compilation of safety impact information for extractables associated with materials used in pharmaceutical packaging, delivery, administration, and manufacturing systems. *PDA J. Pharm. Sci. Technol. 2014 Sep-Oct* ;**68** (5), 407–455. 10.5731/pdajpst.2014.00995 (2024). 10.5731/pdajpst.2014.00995 25336416

[30] Dorato, M. A. & Engelhardt, J. A. The no-observed-adverse-effect-level in drug safety evaluations: use, issues, and definition(s). *Regul. Toxicol. Pharmacol.* **42** (3), 265–274. 10.1016/j.yrtph.2005.05.004 (2005). 15979222 10.1016/j.yrtph.2005.05.004

[31] Ball, D. et al. Development of safety qualification thresholds and their use in orally inhaled and nasal drug product evaluation. *Toxicol. Sci.* **97** (2), 226–236. 10.1093/toxsci/kfm058 (2007). 17369604 10.1093/toxsci/kfm058

[32] Toxicity Estimation Software. Tool (TEST) [Internet] (2024, accessed 25 Mar 2024). https://www.epa.gov/comptox-tools/toxicity-estimation-software-tool-test.

[33] Closset, M. et al. Does an interaction exist between ketamine hydrochloride and Becton Dickinson syringes? *Eur. J. Hosp. Pharm.* **24** (4), 230–234. 10.1136/ejhpharm-2016-001045 (2017). 31156944 10.1136/ejhpharm-2016-001045 PMC6451573

[34] Garay-Aramburu, G. et al. [Translated article] Consensus on intraocular drug preparations. *Farm Hosp.* **49** (2), T99-T108. 10.1016/j.farma.2025.03.004 (2025). 10.1016/j.farma.2025.03.004 40121174

[35] Pascual Carrasco, Á., Espadas García, I., Ramírez López, A. & Selva Otaolaurruchi, J. [Translated article] Syringes for intraocular administration: a systematic review. *Farm. Hosp.* **48** (3), T133-T140. 10.1016/j.farma.2024.04.011 (2024). 10.1016/j.farma.2024.04.011 38705829

[36] McElhiney, L. F. Introduction to hospital compounding. *Int. J. Pharm. Compd.* **10** (4), 245–250 (2006). 23974255

[37] Stucki, C., Sautter, A. M., Wolff, A., Fleury-Souverain, S. & Bonnabry, P. Accuracy of preparation of i.v. medication syringes for anesthesiology. *Am. J. Health Syst. Pharm.* **70** (2), 137–142. 10.2146/ajhp110654 (2013). 23292267 10.2146/ajhp110654

[38] van Gelder, T. G. et al. Drug waste of ready-to-administer syringes in the intensive care unit: aseptically prepared syringes versus prefilled sterilized syringes. *Eur. J. Pharm. Sci.* **191**, 106590. 10.1016/j.ejps.2023.106590 (2023). 37742987 10.1016/j.ejps.2023.106590

[39] Vrignaud, S. Resolution CM/Res(2016)2 and centralised intra venous additive services (CIVAS): challenges and opportunities. *Pharmaceut. Technol. Hospital Pharm.* **2** (3), 137–142. 10.1515/pthp-2017-0023 (2016).

[40] European Parliament and Council. Regulation (EU) 2017/745 of the European Parliament and of the Council of 5 April 2017 on medical devices, and repealing directive 90/385/EEC and directive 93/42/EEC. *Official J. Eur. Union*. **L117**, 1–175 (2017). https://eur-lex.europa.eu/legal-content/EN/TXT/?uri=CELEX%3A32017R0745.

[41] U.S. Food and Drug Administration. Premarket Notification 510(k) Submissions (2023). https://www.fda.gov/medical-devices/premarket-notification-510k.

[42] United States Pharmacopeial Convention. USP < 1663>: assessment of extractables associated with pharmaceutical packaging/delivery systems. *USP–NF*10.31003/USPNF_M7126_03_01 (2024).

[43] United States Pharmacopeial Convention. USP < 1664>: assessment of drug product leachables associated with pharmaceutical packaging/delivery systems. *USP–NF*10.31003/USPNF_M7127_03_01 (2024).

[44] Product Quality Research Institute. (2006–2020). PQRI Leachables and Extractables Working Group Recommendations for Pharmaceutical Drug Products. PQRI (2020). 10.5731/pdajpst.2013.00936 24084659

[45] International Council for Harmonisation of Technical Requirements for Pharmaceuticals for Human Use. ICH Q3E: Concept paper for the development of a guideline for extractables and leachables. Retrieved from ICH website (2020).

[46] European Medicines Agency. *Guideline on Plastic Immediate Packaging Materials (CPMP/QWP/4359/03)* (EMA, 2005).

[47] IARC Working Group on the Evaluation of Carcinogenic Risks to Humans. Some Industrial Chemicals. Lyon (FR): International Agency for Research on Cancer; 2000. (IARC Monographs on the Evaluation of Carcinogenic Risks to Humans, No. 77.) Di(2-ethylhexyl) adipate. https://www.ncbi.nlm.nih.gov/books/NBK390864/ (2000).

[48] Behairy, A., Abd El-Rahman, G. I., Aly, S. S. H., Fahmy, E. M. & Abd-Elhakim, Y. M. Di(2-ethylhexyl) adipate plasticizer triggers hepatic, brain, and cardiac injury in rats: mitigating effect of peganum Harmala oil. *Ecotoxicol. Environ. Saf.* **208**, 111620. 10.1016/j.ecoenv.2020.111620 (2021). 33396140 10.1016/j.ecoenv.2020.111620

[49] Gaëlle, D. et al. Hydrolysis of polydimethylsiloxane fluids in controlled aqueous solutions. *Water Sci. Technol. *. **68** (4), 813–820. 10.2166/wst.2013.308 (2013). 23985511 10.2166/wst.2013.308

[50] Naumoska, K., Jug, U., Metličar, V. & Vovk, I. Oleamide, a bioactive compound, unwittingly introduced into the human body through some plastic food/beverages and medicine containers. *Foods* **9** (5), 549. 10.3390/foods9050549 (2020). 32369935 10.3390/foods9050549 PMC7278760

[51] Jug, U. et al. Interference of oleamide with analytical and bioassay results. *Sci. Rep.* **10** (1), 2163. 10.1038/s41598-020-59093-1 (2020). 32034225 10.1038/s41598-020-59093-1 PMC7005802

[52] Becton & Dickinson and Company. BD Plastipak™ [Technical Sheet]. Consumables Compliance. (n.d.). Technical Data Sheet No. 100–0953 [PDF] (2025). https://downloads.consumables.com/doc/tds/100-0953_TDS.pdf.

[53] Braun Medical, B. AG. Omnifix F ready-to-fill syringe [Product catalog] (2025). https://catalogs.bbraun.ch/fr-CH/p/PRID00000578/omnifix-f?bomUsage=marketingDocuments.

[54] Yilmaz, B., Terekeci, H., Sandal, S. & Kelestimur, F. Endocrine disrupting chemicals: exposure, effects on human health, mechanism of action, models for testing and strategies for prevention. *Rev. Endocr. Metab. Disord*. **21** (1), 127–147. 10.1007/s11154-019-09521-z (2020). 31792807 10.1007/s11154-019-09521-z

[55] Becton Dickinson and Company. BD sterifill Advance™ polymer prefillable syringe for intravenous drugs [Product overview]. *IndiaMART Retrieved July* (2025).

[56] SCHOTT Pharma, A. G. SCHOTT TOPPAC ready-to-use prefillable polymer syringes [Product information] (2025).

[57] Melo, G. B. et al. Release of silicone oil and the off-label use of syringes in ophthalmology. *Br. J. Ophthalmol.* **104** (2), 291–296. 10.1136/bjophthalmol-2019-313823 (2020). 30910872 10.1136/bjophthalmol-2019-313823

