# Supplementary material for: Assessment of leachables in hospital pharmacy compounded topotecan conditioned in common off-label syringes for intravitreal use
Source: Sci Rep. 2025 Nov 19;15:40736. doi: 10.1038/s41598-025-24557-9 (PMC12630713; doi:10.1038/s41598-025-24557-9)
Supplement: Supplementary file 1 — Supplementary Material 1 [file 41598_2025_24557_MOESM1_ESM.docx]

**ELECTRONIC SUPPLEMENTARY INFORMATION**

**Assessment of Leachables in Hospital Pharmacy Compounded Topotecan Conditioned in Common Off-Label Syringes for Intravitreal Use**

# **AUTHORS**: William Bello^1,2,3,4^, Camille Hosotte^1,2,3,4^, Camille Stampfli^1^, Antoine Pierrot^1^, Francis L. Munier^5^, Markoulina Berger-Gryllaki^1^, Laurent Carrez^1^, Julian Pezzatti^1^, Farshid Sadeghipour^1,2,3,4^*

1. Pharmacy Department, Lausanne University Hospital
2. School of Pharmaceutical Sciences, University of Geneva, CMU-Rue Michel Servet 1, 1211, Geneva 4, Switzerland
3. Institute of Pharmaceutical Sciences of Western Switzerland, University of Geneva, University of Lausanne
4. Center for Research and Innovation in Clinical Pharmaceutical Sciences, Lausanne University Hospital and University of Lausanne
5. Jules-Gonin Eye Hospital, Fondation Asile des Aveugles, University of Lausanne, Lausanne, Switzerland.

*Corresponding author

# CORRESPONDENCE:

Prof. F. Sadeghipour, Pharmacy Department, Lausanne University Hospital, Switzerland; Centre for Research and Innovation in Clinical Pharmaceutical Sciences, Lausanne University Hospital and University of Lausanne, Switzerland ; School of Pharmaceutical Sciences, University of Geneva, CMU-Rue Michel Servet 1, 1211, Geneva 4, Switzerland ; Institute of Pharmaceutical Sciences of Western Switzerland, University of Geneva, CMU-Rue Michel Servet 1, 1211, Geneva 4, Switzerland

E-mail: farshid.sadeghipour@unige.ch; farshid.sadeghipour@chuv.ch

# ORCID

William Bello 0009-0009-8704-130X

Camille Hosotte 0009-0002-6680-0062

Camille Stampfli 0000-0002-6659-0790

Antoine Pierrot 0009-0001-7937-611X

Francis Munier 0000-0002-8928-1050

Markoulina Berger-Gryllaki 0009-0004-1833-9616

Laurent Carrez 0000-0002-6546-8128

Julian Pezzatti 0000-0002-3329-8240

Farshid Sadeghipour 0000-0003-0817-5393

**Table S1.** Compilation of chromatographic and mass spectrometric data on all 24 plastic-related compounds identified in the hospital pharmacy compounded drug products containing Topotecan (both BD Plastipak and BBraun Omnifix).

| N° | Plastic Additives | Product code | CAS | Additive groups | Chemical Formula | Polarity | Charge | Monoisotopic mass | Retention time (minutes) | Mass [m/z] |
| --- | --- | --- | --- | --- | --- | --- | --- | --- | --- | --- |
| 1 | 2,6-di-tert-butyl-4-hydroxy-4-methylcyclohexa-2,5-dien-1-one | AO120 | 10396-80-2 | Antioxidants | C15H24O2 | negative | +H | 236.177628 | 3.508 | 235.17035 |
| 2 | 2,6-di-tert-butyl-p-cresol | AO95 | 128-37-0 | Antioxidants | C15H24O | negative | +H | 220.182709 | 5.26 | 219.17544 |
| 3 | 3-(3,5-di-tert-butyl-4-hydroxyphenyl)propanoic acid | AO26 | 20170-32-5 | Antioxidants | C17H26O3 | negative | +H | 278.188202 | 3.52 | 277.18092 |
| 4 | 3-(3-5-di-tert-butyl-1-hydroxy-4-oxo-2,5-cyclohexadiene-1-yl)propanoic acid | AO45 | 83237-15-4 | Antioxidants | C17H26O4 | negative | +H | 294.183105 | 2.636 | 293.17583 |
| 5 | 3,5-di-tert-butyl-4-hydroxybenzaldehyde | AO25 | 1620-98-0 | Antioxidants | C15H22O2 | negative | +H | 234.161987 | 3.82 | 233.1547 |
| 6 | 3,5-Di-tert-butyl-4-hydroxybenzyl alcohol | AO48 | 88-26-6 | Antioxidants | C15H24O2 | negative | +H | 236.177628 | 3.13 | 235.17035 |
| 7 | Acetyl tributyl citrate | PZ17 | 77-90-7 | Plasticizers | C20H34O8 | positive | +H | 402.225372 | 5.384 | 403.23264 |
| 8 | Benzotriazole | UV25 | 95-14-7 | UV Stabilizers | C6H5N3 | positive | +H | 119.048347 | 2.08 | 120.05562 |
| 9 | Bisphenol A | BP14 | 80-05-7 | Bisphenols | C15H16O2 | negative | +H | 228.115036 | 2.38 | 227.10775 |
| 10 | Caprolactam | O1 | 105-60-2 | Miscellaneous | C6H11NO | positive | +H | 113.084061 | 2.2 | 114.09134 |
| 11 | Di-(2-ethylhexyl) adipate | PZ2 | 103-23-1 | Plasticizers | C22H42O4 | positive | +H | 370.308319 | 8.69 | 371.31559 |
| 12 | Di-(2-ethylhexyl) sebacate | PZ104 | 122-62-3 | Plasticizers | C26H50O4 | positive | +NH4 | 426.370911 | 10.24 | 444.40474 |
| 13 | N,N-Dibutylformamide | RA6 | 761-65-9 | Miscellaneous | C9H19NO | positive | +H | 157.146667 | 2.85 | 158.15394 |
| 14 | N-butylformamide | RA9 | 111-36-4 | Miscellaneous | C5H11NO | positive | +H | 101.084061 | 2.018 | 102.09134 |
| 15 | Oleamide | LB1 | 301-02-0 | Miscellaneous | C18H35NO | positive | +H | 281.271851 | 6.6 | 282.27914 |
| 16 | Tetradecanamide | LB3 | 638-58-4 | Miscellaneous | C14H29NO | positive | +H | 227.224915 | 4.8 | 228.23219 |
| 17 | triethyleneglycol dimethacrylate | AD16 | 109-16-0 | Miscellaneous | C14H22O6 | positive | +H | 286.141632 | 2.91 | 304.17546 |
| 18 | Triphenyl phosphate | PZ36 | 115-86-6 | Plasticizers | C18H15O4P | positive | +H | 326.070801 | 3.95 | 327.07807 |
| 19 | Tris ( 2-chloro-1-methylethyl ) phosphate | PZ39 | 13674-84-5 | Plasticizers | C9H18Cl3O4P | positive | +H | 326.000824 | 3.16 | 327.00811 |
| 20 | hexamethylcyclotrisiloxane | LB4 | 541-05-9 | Miscellaneous | C6H18O3Si3 | positive | +H | 222.056366 | 2.5 | 223.06365 |
| 21 | octamethylcyclotetrasiloxane | LB5 | 556-67-2 | Miscellaneous | C8H24O4Si4 | positive | +H | 296.075165 | 3.25 | 297.08244 |
| 22 | decamethyltetrasiloxane | LB6 | 141-62-8 | Miscellaneous | C10H30O3Si4 | positive | +H | 310.127197 | 4.83 | 311.13448 |
| 23 | decamethylcyclopentasiloxane | LB7 | 541-02-6 | Miscellaneous | C10H30O5Si5 | positive | +H | 370.093964 | 7.33 | 371.10123 |
| 24 | dodecamethylpentasiloxane | LB8 | 141-63-9 | Miscellaneous | C12H36O4Si5 | positive | +H | 384.145996 | 8.39 | 385.15327 |

**Table S2.** Compilation of semiquantitative results on all 24 plastic-related compounds identified in hospital pharmacy compounding topotecan prefilled syringes conditioned in both BD Plastipak and BBraun Omnifix after 12 months of storage. Table cells coloured in grey shows no values.

| N° | Plastic Additives | Product code | CAS | Additive groups | Chemical Formula | Polarity | Charge | LOD (Concentration in ng/mL) | LOQ (Concentration in ng/mL) | Topotecan PFS BD Plastipak (Concentration in ng/mL) | Topotecan PFS BBraun Omnifix  (Concentration in ng/mL) |
| --- | --- | --- | --- | --- | --- | --- | --- | --- | --- | --- | --- |
| 1 | 2,6-di-tert-butyl-4-hydroxy-4-methylcyclohexa-2,5-dien-1-one | AO120 | 10396-80-2 | Antioxidants | C15H24O2 | negative | +H | 0.12 | 0.37 | 0.31 |  |
| 2 | 2,6-di-tert-butyl-p-cresol | AO95 | 128-37-0 | Antioxidants | C15H24O | negative | +H | 0.98 | 2.93 | 0.17 | 0.23 |
| 3 | 3-(3,5-di-tert-butyl-4-hydroxyphenyl)propanoic acid | AO26 | 20170-32-5 | Antioxidants | C17H26O3 | negative | +H | 0.12 | 0.37 | 6.3 | 2.4 |
| 4 | 3-(3-5-di-tert-butyl-1-hydroxy-4-oxo-2,5-cyclohexadiene-1-yl)propanoic acid | AO45 | 83237-15-4 | Antioxidants | C17H26O4 | negative | +H | 0.12 | 0.37 |  | 6.2 |
| 5 | 3,5-di-tert-butyl-4-hydroxybenzaldehyde | AO25 | 1620-98-0 | Antioxidants | C15H22O2 | negative | +H | 0.06 | 0.18 | 0.55 | 1.2 |
| 6 | 3,5-Di-tert-butyl-4-hydroxybenzyl alcohol | AO48 | 88-26-6 | Antioxidants | C15H24O2 | negative | +H | 0.12 | 0.37 |  | 1.3 |
| 7 | Acetyl tributyl citrate | PZ17 | 77-90-7 | Plasticizers | C20H34O8 | positive | +H | 0.12 | 0.36 | 3.2 | 4.7 |
| 8 | Benzotriazole | UV25 | 95-14-7 | UV Stabilizers | C6H5N3 | positive | +H | 0.12 | 0.36 | 0.95 |  |
| 9 | Bisphenol A | BP14 | 80-05-7 | Bisphenols | C15H16O2 | negative | +H | 0.49 | 1.46 | 0.32 |  |
| 10 | Caprolactam | O1 | 105-60-2 | Miscellaneous | C6H11NO | positive | +H | 0.24 | 0.73 | 0.62 | 0.37 |
| 11 | Di-(2-ethylhexyl) adipate | PZ2 | 103-23-1 | Plasticizers | C22H42O4 | positive | +H | 0.49 | 1.46 | 15.1 | 4.7 |
| 12 | Di-(2-ethylhexyl) sebacate | PZ104 | 122-62-3 | Plasticizers | C26H50O4 | positive | +NH4 | 0.98 | 2.93 | 0.64 |  |
| 13 | N,N-Dibutylformamide | RA6 | 761-65-9 | Miscellaneous | C9H19NO | positive | +H | 0.24 | 0.73 | 0.94 |  |
| 14 | N-butylformamide | RA9 | 111-36-4 | Miscellaneous | C5H11NO | positive | +H | 1.95 | 5.86 | 0.65 |  |
| 15 | Oleamide | LB1 | 301-02-0 | Miscellaneous | C18H35NO | positive | +H | 0.12 | 0.37 |  | 24.7 |
| 16 | Tetradecanamide | LB3 | 638-58-4 | Miscellaneous | C14H29NO | positive | +H | 0.12 | 0.37 |  | 6.3 |
| 17 | triethyleneglycol dimethacrylate | AD16 | 109-16-0 | Miscellaneous | C14H22O6 | positive | +H | 0.12 | 0.37 |  | 0.47 |
| 18 | Triphenyl phosphate | PZ36 | 115-86-6 | Plasticizers | C18H15O4P | positive | +H | 0.03 | 0.09 | 0.21 | 0.12 |
| 19 | Tris ( 2-chloro-1-methylethyl ) phosphate | PZ39 | 13674-84-5 | Plasticizers | C9H18Cl3O4P | positive | +H | 0.06 | 0.183 | 7.6 | 0.149 |
| 20 | hexamethylcyclotrisiloxane | LB4 | 541-05-9 | Miscellaneous | C6H18O3Si3 | positive | +H | 0.97 | 2.91 | 67 | 3.57 |
| 21 | octamethylcyclotetrasiloxane | LB5 | 556-67-2 | Miscellaneous | C8H24O4Si4 | positive | +H | 3.91 | 11.72 | 27 |  |
| 22 | decamethyltetrasiloxane | LB6 | 141-62-8 | Miscellaneous | C10H30O3Si4 | positive | +H | 7.81 | 23.43 | 43 |  |
| 23 | decamethylcyclopentasiloxane | LB7 | 541-02-6 | Miscellaneous | C10H30O5Si5 | positive | +H | 62.5 | 187.5 | 231 |  |
| 24 | dodecamethylpentasiloxane | LB8 | 141-63-9 | Miscellaneous | C12H36O4Si5 | positive | +H | 62.5 | 187.5 | 333 |  |

**Table S3.** Compilation of toxicological data, basing it on the MDD of the drug product and the weight of supposed patient, on all 24 plastic-related compounds identified in hospital pharmacy compounding topotecan prefilled syringes conditioned in BD Plastipak, including the TDE and the PDE after 12 months of storage. Pink salmon coloured cells are supposedly potential endocrine disruptors. Table cells coloured in grey shows no values.

| N° | Plastic Additives | Product code | CAS | TDE TPT PFS (10kg) (MDD = 1mL) (ng/day) | TDE TPT PFS (20kg) (MDD = 1mL) (ng/day) | PDE (est) - ng/kg bw/day | PDE (est) - ng/day for a patient of 10kg | PDE (est) - ng/day for a patient of 20kg | PDE (est) - ng/day for a patient of 10kg | PDE (est) - ng/day for a patient of 20kg |
| --- | --- | --- | --- | --- | --- | --- | --- | --- | --- | --- |
| 1 | 2,6-di-tert-butyl-4-hydroxy-4-methylcyclohexa-2,5-dien-1-one | AO120 | 10396-80-2 | 3.1 | 6.2 | 94.66 | 946.55 | 1893.10 | 2.98 | 3.28 |
| 2 | 2,6-di-tert-butyl-p-cresol | AO95 | 128-37-0 | 1.7 | 3.4 | 71.26 | 712.63 | 1425.25 | 2.85 | 3.15 |
| 3 | 3-(3,5-di-tert-butyl-4-hydroxyphenyl)propanoic acid | AO26 | 20170-32-5 | 63 | 126 | 92.18 | 921.85 | 1843.69 | 2.96 | 3.27 |
| 4 | 3-(3-5-di-tert-butyl-1-hydroxy-4-oxo-2,5-cyclohexadiene-1-yl)propanoic acid | AO45 | 83237-15-4 |  |  | 14.39 | 143.94 | 287.88 | 2.16 | 2.46 |
| 5 | 3,5-di-tert-butyl-4-hydroxybenzaldehyde | AO25 | 1620-98-0 | 5.5 | 11 | 128.94 | 1289.42 | 2578.84 | 3.11 | 3.41 |
| 6 | 3,5-Di-tert-butyl-4-hydroxybenzyl alcohol | AO48 | 88-26-6 |  |  | 81.70 | 816.99 | 1633.97 | 2.91 | 3.21 |
| 7 | Acetyl tributyl citrate | PZ17 | 77-90-7 | 32 | 64 | 582.94 | 5829.44 | 11658.87 | 3.77 | 4.07 |
| 8 | Benzotriazole | UV25 | 95-14-7 | 9.5 | 19 | 26.13 | 261.27 | 522.54 | 2.42 | 2.72 |
| 9 | Bisphenol A | BP14 | 80-05-7 | 3.2 | 6.4 | 205.45 | 2054.50 | 4108.99 | 3.31 | 3.61 |
| 10 | Caprolactam | O1 | 105-60-2 | 6.2 | 12.4 | 104.00 | 1040.01 | 2080.01 | 3.02 | 3.32 |
| 11 | Di-(2-ethylhexyl) adipate | PZ2 | 103-23-1 | 151 | 302 | 484.80 | 4847.96 | 9695.92 | 3.69 | 3.99 |
| 12 | Di-(2-ethylhexyl) sebacate | PZ104 | 122-62-3 | 6.4 | 12.8 | 677.49 | 6774.88 | 13549.76 | 3.83 | 4.13 |
| 13 | N,N-Dibutylformamide | RA6 | 761-65-9 | 9.4 | 18.8 | 51.45 | 514.47 | 1028.93 | 2.71 | 3.01 |
| 14 | N-butylformamide | RA9 | 111-36-4 | 6.5 | 13 | 17.16 | 171.61 | 343.22 | 2.23 | 2.54 |
| 15 | Oleamide | LB1 | 301-02-0 |  |  | 316.36 | 3163.64 | 6327.28 | 3.50 | 3.80 |
| 16 | Tetradecanamide | LB3 | 638-58-4 |  |  | 109.25 | 1092.55 | 2185.09 | 3.04 | 3.34 |
| 17 | triethyleneglycol dimethacrylate | AD16 | 109-16-0 |  |  | 360.46 | 3604.62 | 7209.24 | 3.56 | 3.86 |
| 18 | Triphenyl phosphate | PZ36 | 115-86-6 | 2.1 | 4.2 | 113.54 | 1135.41 | 2270.81 | 3.06 | 3.36 |
| 19 | Tris ( 2-chloro-1-methylethyl ) phosphate | PZ39 | 13674-84-5 | 76 | 152 | 20.50 | 204.99 | 409.97 | 2.31 | 2.61 |
| 20 | hexamethylcyclotrisiloxane | LB4 | 541-05-9 | 670 | 1340 | 70.69 | 706.89 | 1413.77 | 2.85 | 3.15 |
| 21 | octamethylcyclotetrasiloxane | LB5 | 556-67-2 | 270 | 540 | 54.73 | 547.34 | 1094.67 | 2.74 | 3.04 |
| 22 | decamethyltetrasiloxane | LB6 | 141-62-8 | 430 | 860 | 184.28 | 1842.81 | 3685.62 | 3.27 | 3.57 |
| 23 | decamethylcyclopentasiloxane | LB7 | 541-02-6 | 2310 | 4620 | 184.28 | 1842.81 | 3685.62 | 3.27 | 3.57 |
| 24 | dodecamethylpentasiloxane | LB8 | 141-63-9 | 3330 | 6660 | 184.28 | 1842.81 | 3685.62 | 3.27 | 3.57 |

**Table S4.** Compilation of toxicological data, basing it on the MDD of the drug product and the weight of supposed patient, on all 24 plastic-related compounds identified in hospital pharmacy compounding topotecan prefilled syringes conditioned in BBraun Omnifix, including the TDE and the PDE, after 12 months of storage. Pink salmon coloured cells are supposedly potential endocrine disruptors. Table cells coloured in grey shows no values.

| N° | Plastic Additives | Product code | CAS | TDE TPT PFS (10kg) (MDD = 1mL) (ng/day) | TDE TPT PFS (20kg) (MDD = 1mL) (ng/day) | PDE (est) - ng/kg bw/day | PDE (est) - ng/day for a patient of 10kg | PDE (est) - ng/day for a patient of 20kg | PDE (est) - ng/day for a patient of 10kg | PDE (est) - ng/day for a patient of 20kg |
| --- | --- | --- | --- | --- | --- | --- | --- | --- | --- | --- |
| 1 | 2,6-di-tert-butyl-4-hydroxy-4-methylcyclohexa-2,5-dien-1-one | AO120 | 10396-80-2 |  |  | 94.66 | 946.55 | 1893.10 | 2.98 | 3.28 |
| 2 | 2,6-di-tert-butyl-p-cresol | AO95 | 128-37-0 | 2.30 | 4.60 | 71.26 | 712.63 | 1425.25 | 2.85 | 3.15 |
| 3 | 3-(3,5-di-tert-butyl-4-hydroxyphenyl)propanoic acid | AO26 | 20170-32-5 | 24.00 | 48.00 | 92.18 | 921.85 | 1843.69 | 2.96 | 3.27 |
| 4 | 3-(3-5-di-tert-butyl-1-hydroxy-4-oxo-2,5-cyclohexadiene-1-yl)propanoic acid | AO45 | 83237-15-4 | 62.00 | 124.00 | 14.39 | 143.94 | 287.88 | 2.16 | 2.46 |
| 5 | 3,5-di-tert-butyl-4-hydroxybenzaldehyde | AO25 | 1620-98-0 | 12.00 | 24.00 | 128.94 | 1289.42 | 2578.84 | 3.11 | 3.41 |
| 6 | 3,5-Di-tert-butyl-4-hydroxybenzyl alcohol | AO48 | 88-26-6 | 13.00 | 26.00 | 81.70 | 816.99 | 1633.97 | 2.91 | 3.21 |
| 7 | Acetyl tributyl citrate | PZ17 | 77-90-7 | 47.00 | 94.00 | 582.94 | 5829.44 | 11658.87 | 3.77 | 4.07 |
| 8 | Benzotriazole | UV25 | 95-14-7 |  |  | 26.13 | 261.27 | 522.54 | 2.42 | 2.72 |
| 9 | Bisphenol A | BP14 | 80-05-7 |  |  | 205.45 | 2054.50 | 4108.99 | 3.31 | 3.61 |
| 10 | Caprolactam | O1 | 105-60-2 | 3.70 | 7.40 | 104.00 | 1040.01 | 2080.01 | 3.02 | 3.32 |
| 11 | Di-(2-ethylhexyl) adipate | PZ2 | 103-23-1 | 47.00 | 94.00 | 484.80 | 4847.96 | 9695.92 | 3.69 | 3.99 |
| 12 | Di-(2-ethylhexyl) sebacate | PZ104 | 122-62-3 |  |  | 677.49 | 6774.88 | 13549.76 | 3.83 | 4.13 |
| 13 | N,N-Dibutylformamide | RA6 | 761-65-9 |  |  | 51.45 | 514.47 | 1028.93 | 2.71 | 3.01 |
| 14 | N-butylformamide | RA9 | 111-36-4 |  |  | 17.16 | 171.61 | 343.22 | 2.23 | 2.54 |
| 15 | Oleamide | LB1 | 301-02-0 | 247.00 | 494.00 | 316.36 | 3163.64 | 6327.28 | 3.50 | 3.80 |
| 16 | Tetradecanamide | LB3 | 638-58-4 | 63.00 | 126.00 | 109.25 | 1092.55 | 2185.09 | 3.04 | 3.34 |
| 17 | triethyleneglycol dimethacrylate | AD16 | 109-16-0 | 4.70 | 9.40 | 360.46 | 3604.62 | 7209.24 | 3.56 | 3.86 |
| 18 | Triphenyl phosphate | PZ36 | 115-86-6 | 1.20 | 2.40 | 113.54 | 1135.41 | 2270.81 | 3.06 | 3.36 |
| 19 | Tris ( 2-chloro-1-methylethyl ) phosphate | PZ39 | 13674-84-5 | 1.49 | 2.98 | 20.50 | 204.99 | 409.97 | 2.31 | 2.61 |
| 20 | hexamethylcyclotrisiloxane | LB4 | 541-05-9 | 35.70 | 71.40 | 70.69 | 706.89 | 1413.77 | 2.85 | 3.15 |
| 21 | octamethylcyclotetrasiloxane | LB5 | 556-67-2 |  |  | 54.73 | 547.34 | 1094.67 | 2.74 | 3.04 |
| 22 | decamethyltetrasiloxane | LB6 | 141-62-8 |  |  | 184.28 | 1842.81 | 3685.62 | 3.27 | 3.57 |
| 23 | decamethylcyclopentasiloxane | LB7 | 541-02-6 |  |  | 184.28 | 1842.81 | 3685.62 | 3.27 | 3.57 |
| 24 | dodecamethylpentasiloxane | LB8 | 141-63-9 |  |  | 184.28 | 1842.81 | 3685.62 | 3.27 | 3.57 |


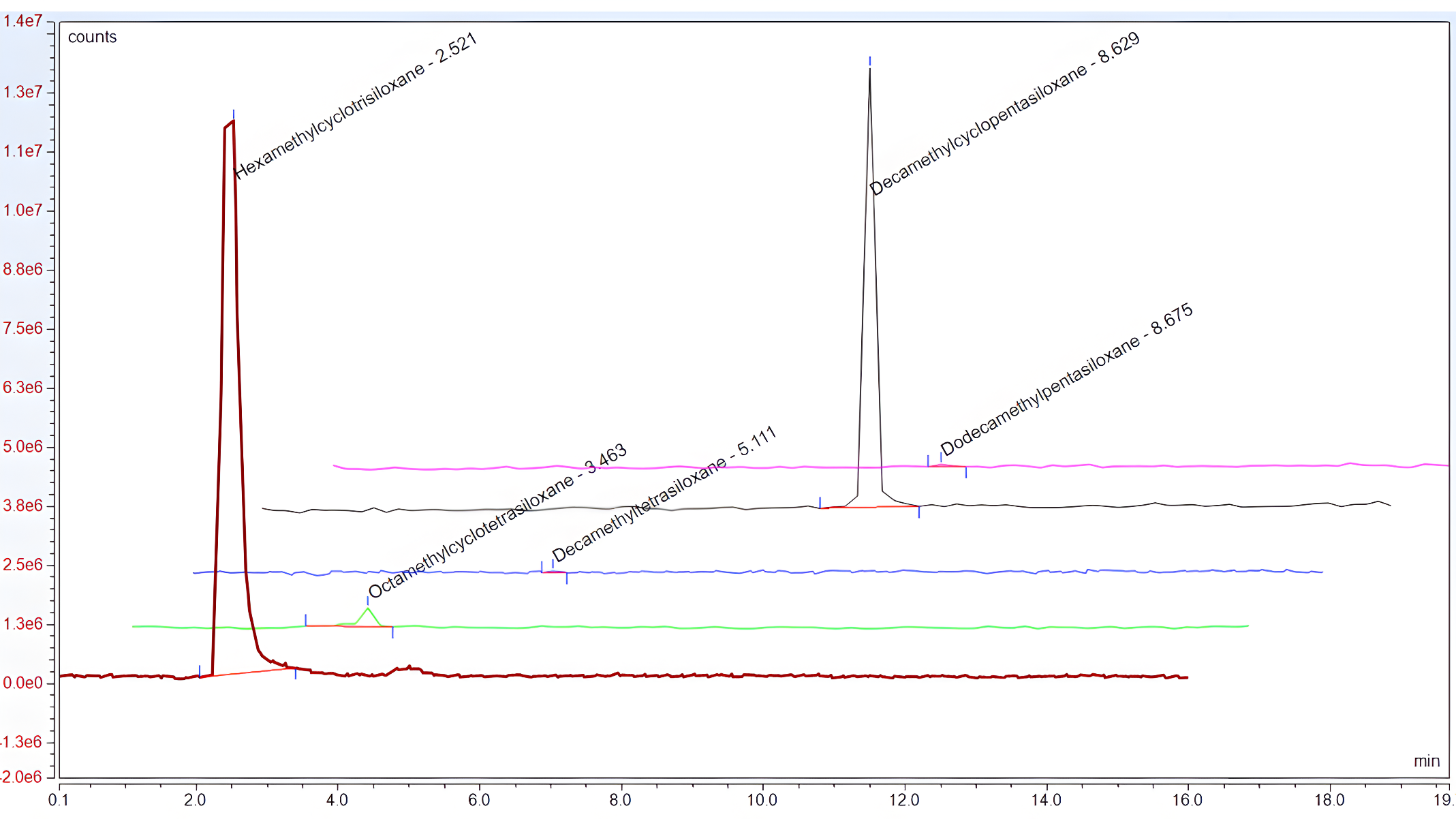


**Fig. S1.** Compiled chromatograms detected in positive mode showing all silicone derivatives observed in BD Plastipak after 12 months storage.

**
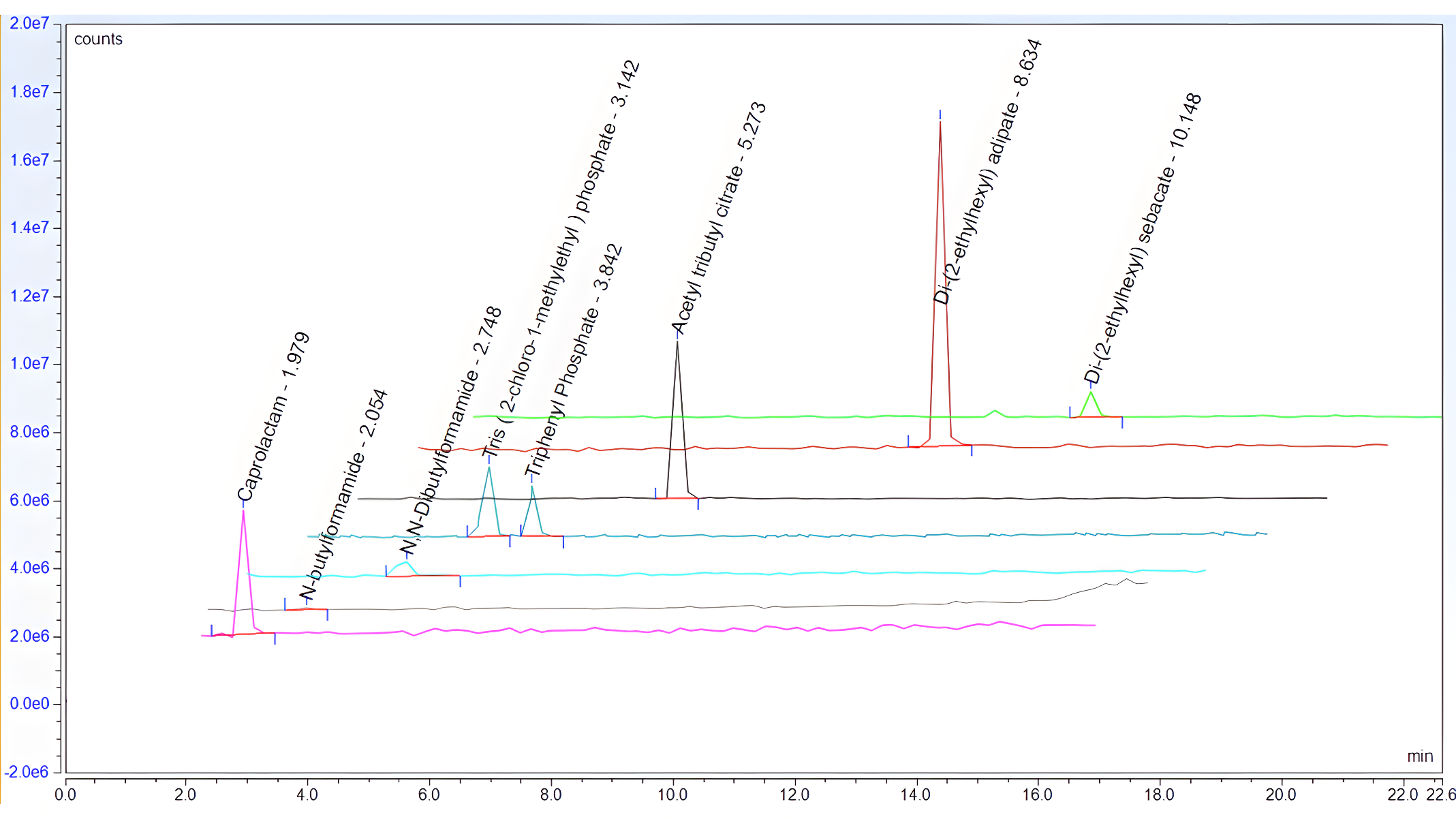
**

**Fig. S2.** Compiled chromatograms detected in positive mode showing other leachable compounds observed in BD Plastipak after 12 months storage.

**
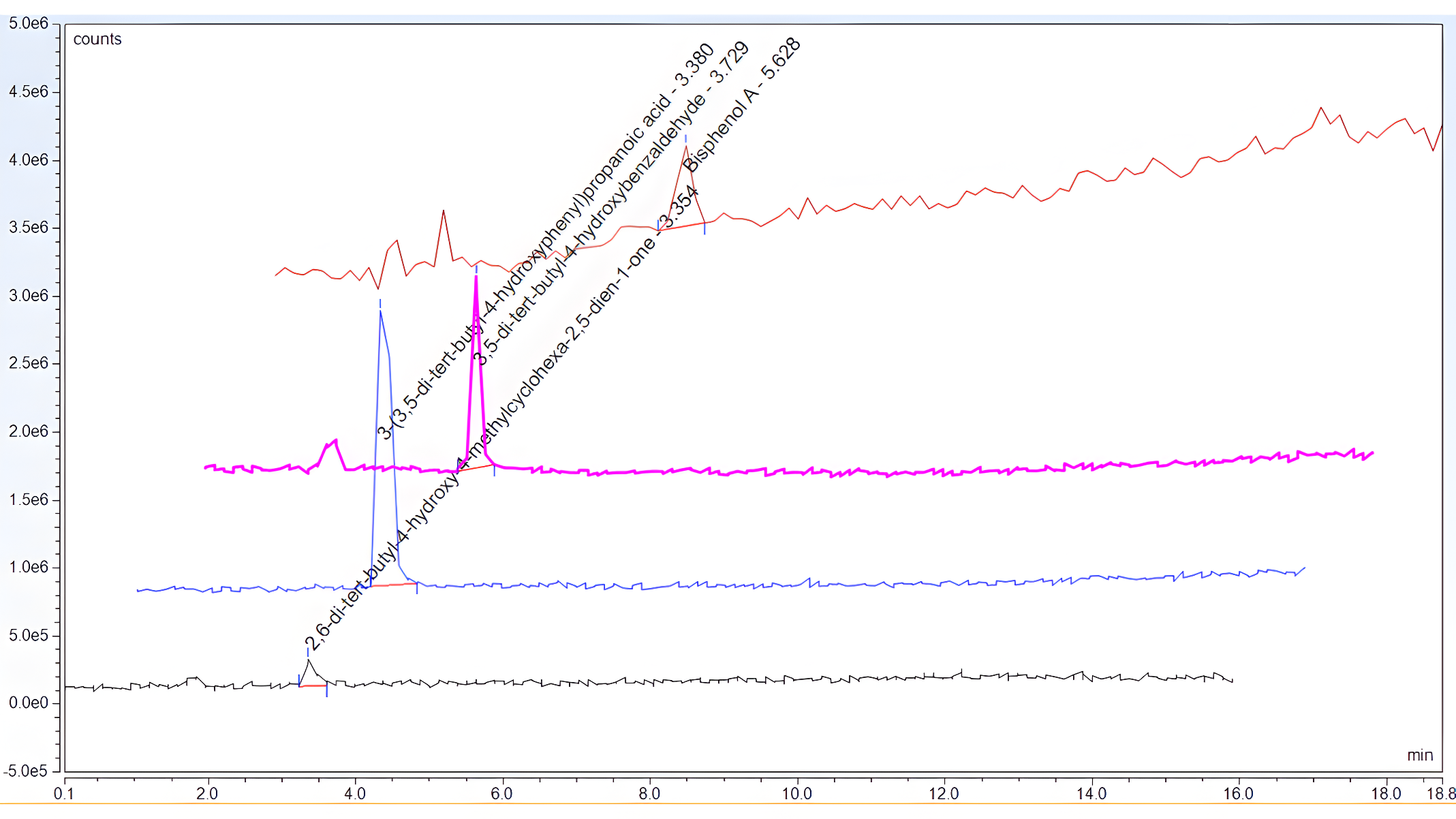
**

**Fig. S3.** Compiled chromatograms detected in negative mode showing other leachable compounds observed in BD Plastipak after 12 months storage.

**
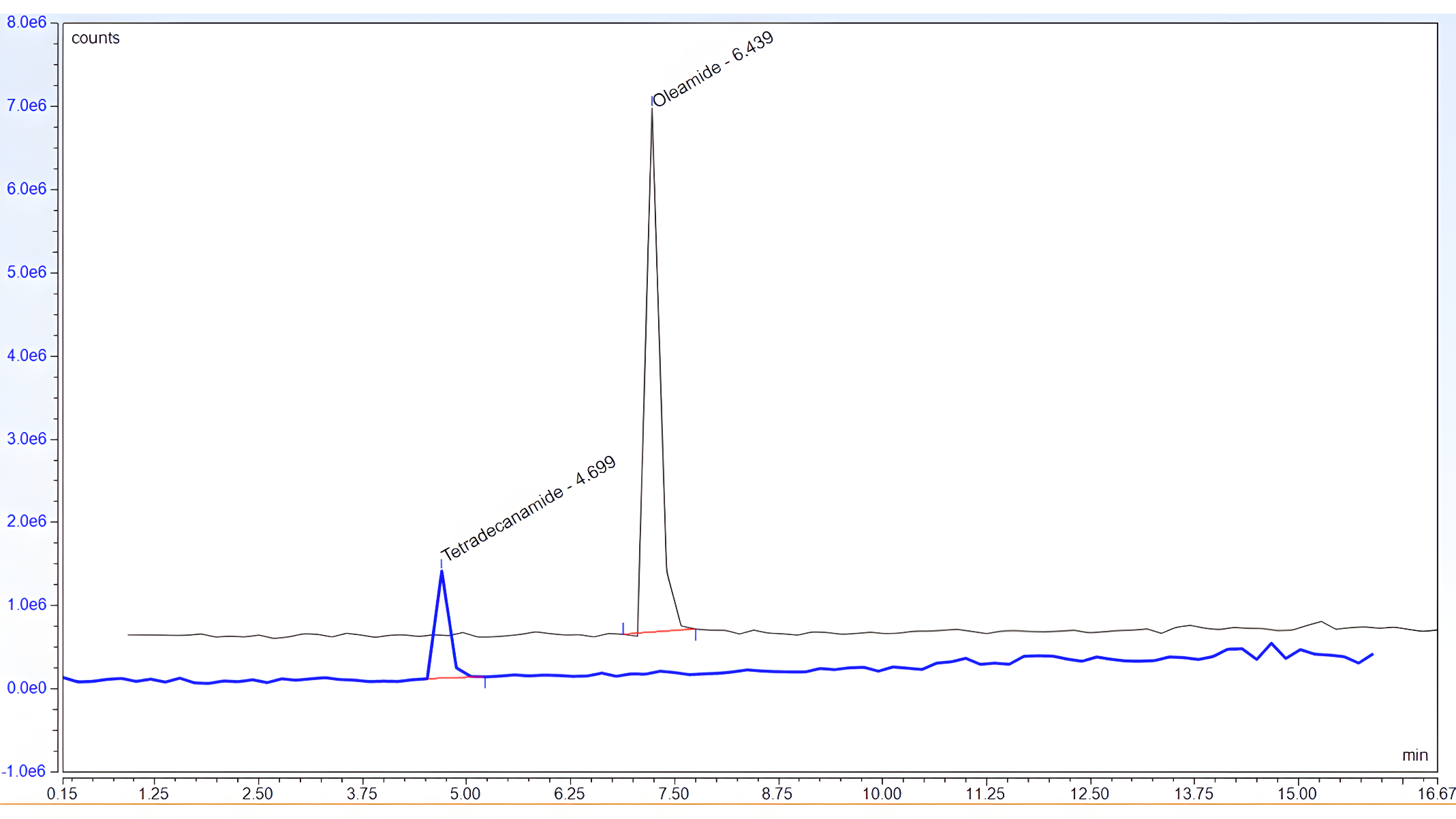
**

**Fig. S4.** Compiled chromatograms detected in positive mode showing Oleamide and its derivative, tetradecanamide, observed in BBraun Omnifix after 12 months storage.

**
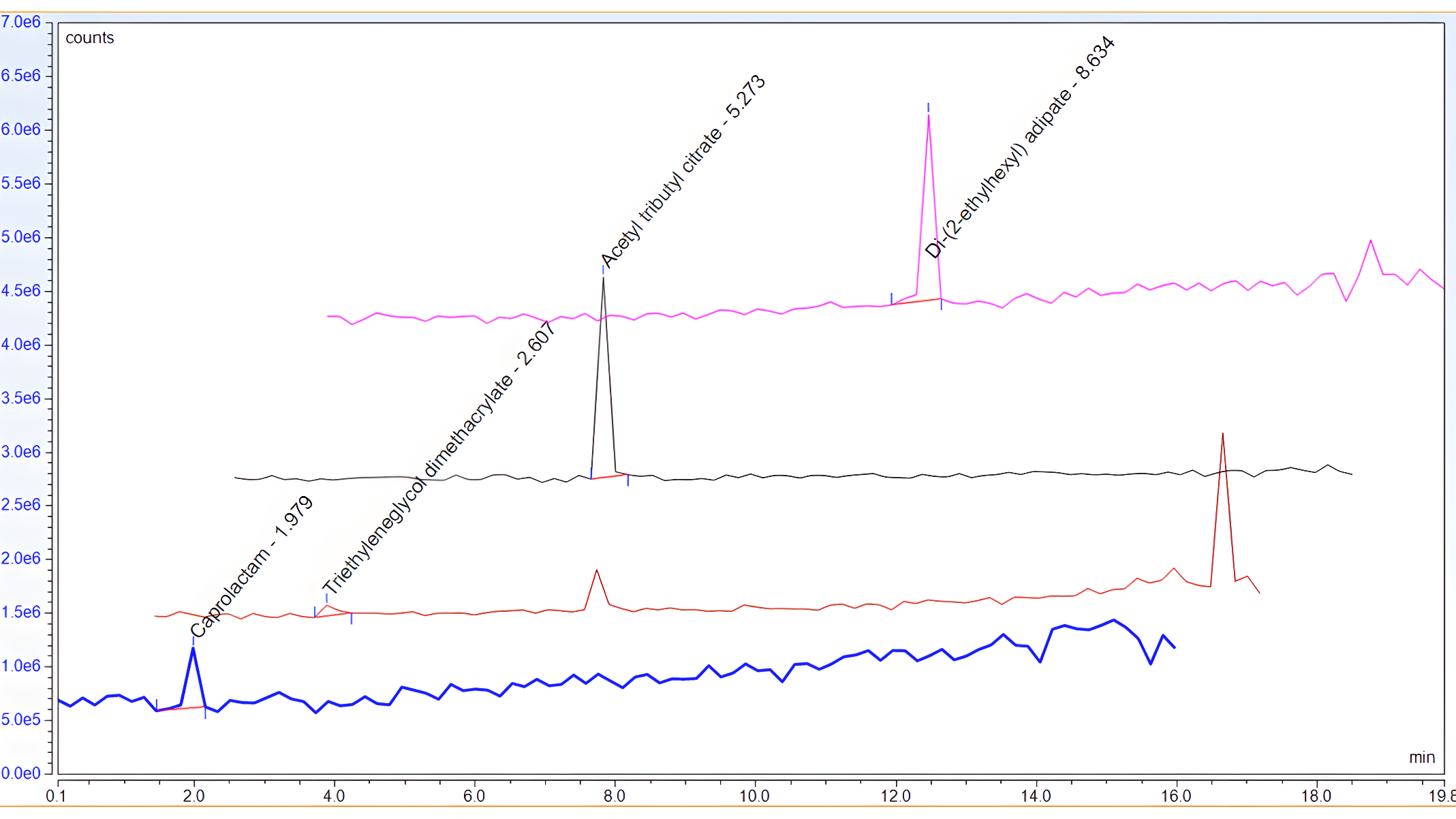
Fig. S5.** Compiled chromatograms detected in positive mode showing other leachable compounds, observed in BBraun Omnifix after 12 months storage.

**
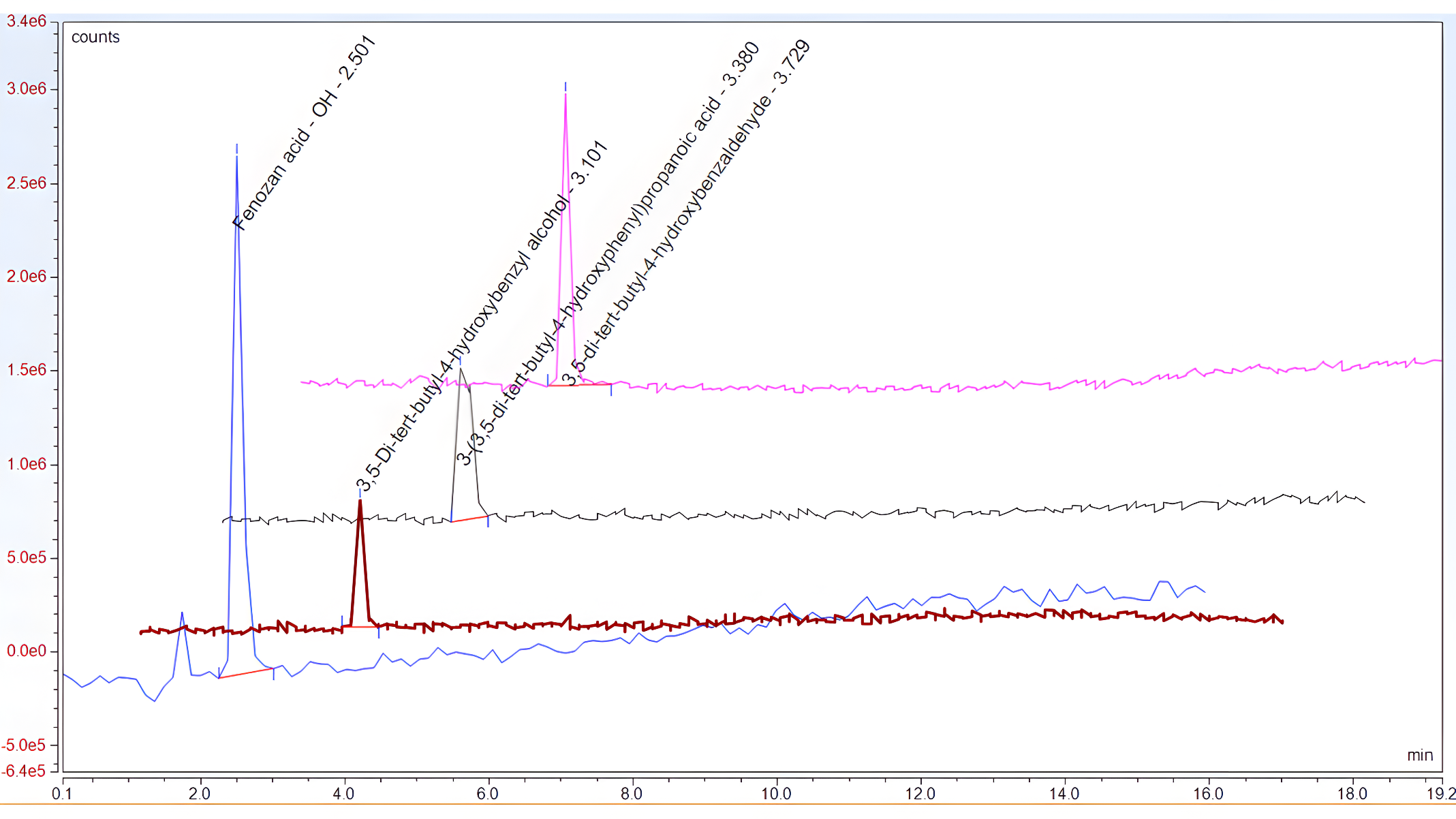
**

**Fig. S6.** Compiled chromatograms detected in negative mode showing other leachable compounds, observed in BBraun Omnifix after 12 months storage.

**
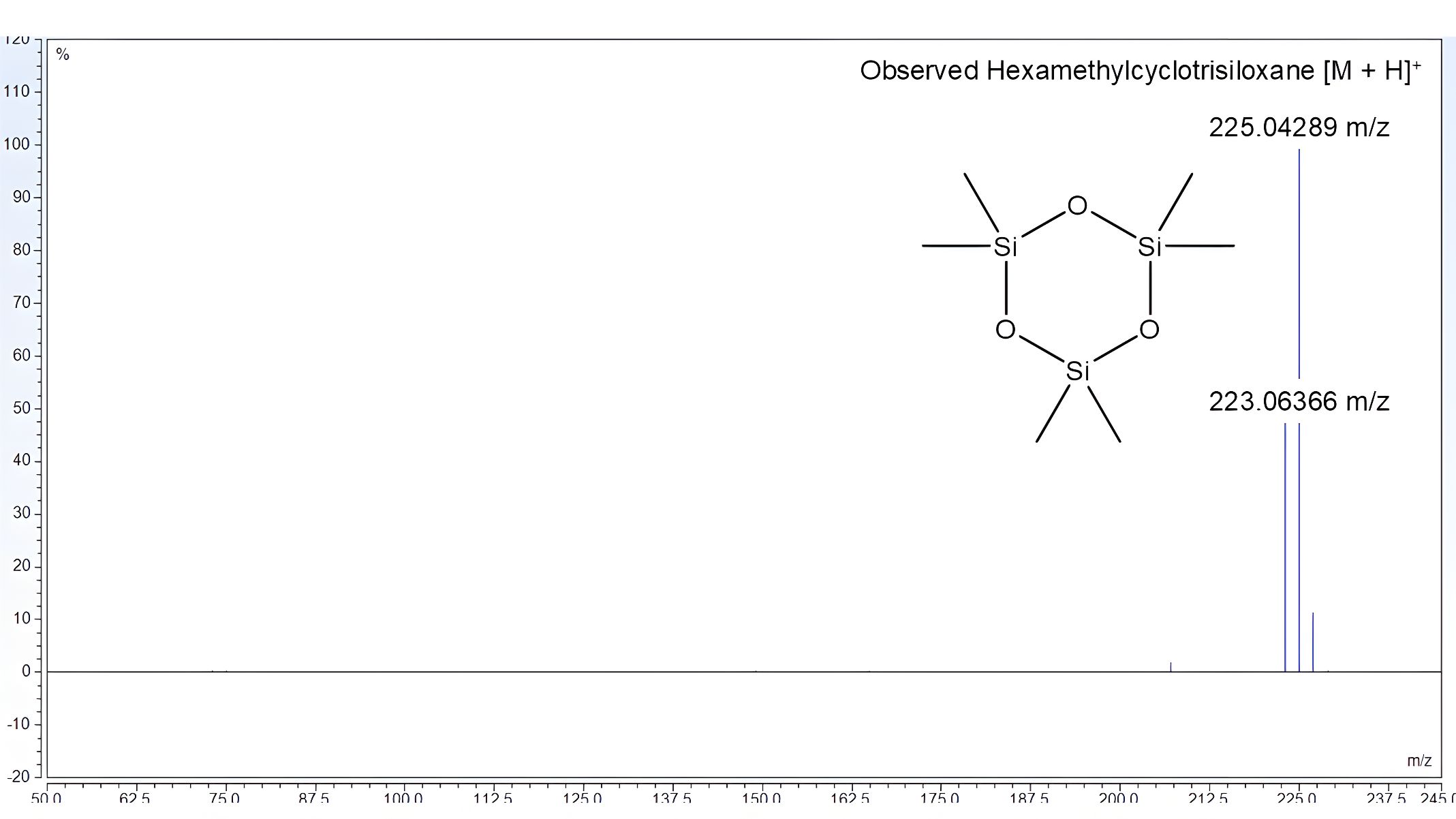
**

**Fig. S7.** Example of mass spectrum of an observed Hexamethylcyclotrisiloxane in BD Plastipak in positive mode**.**

**
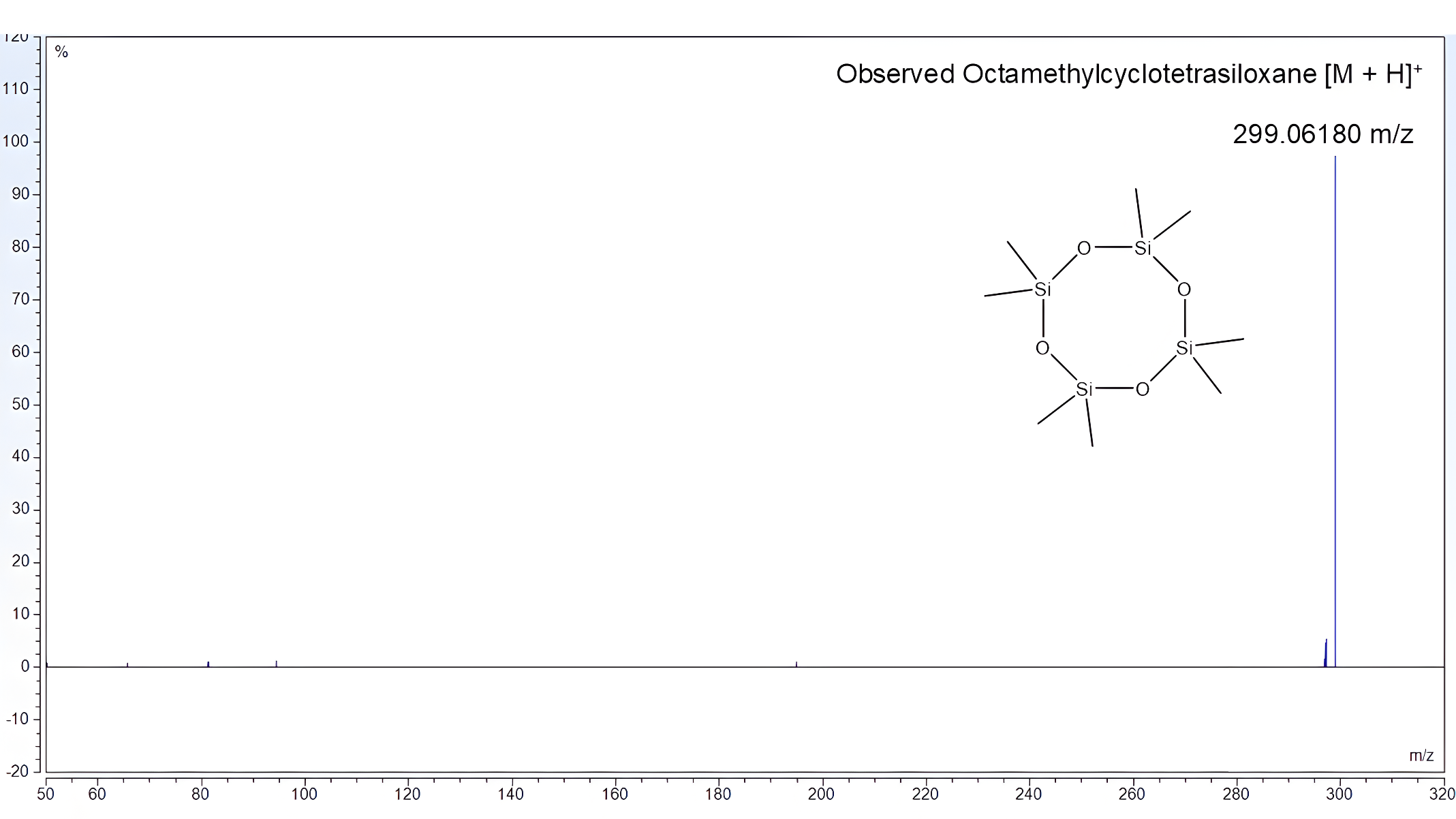
**

**Fig. S8.** Example of mass spectrum of Octamethylcyclotetrasiloxane in BD Plastipak in positive mode**.**

**
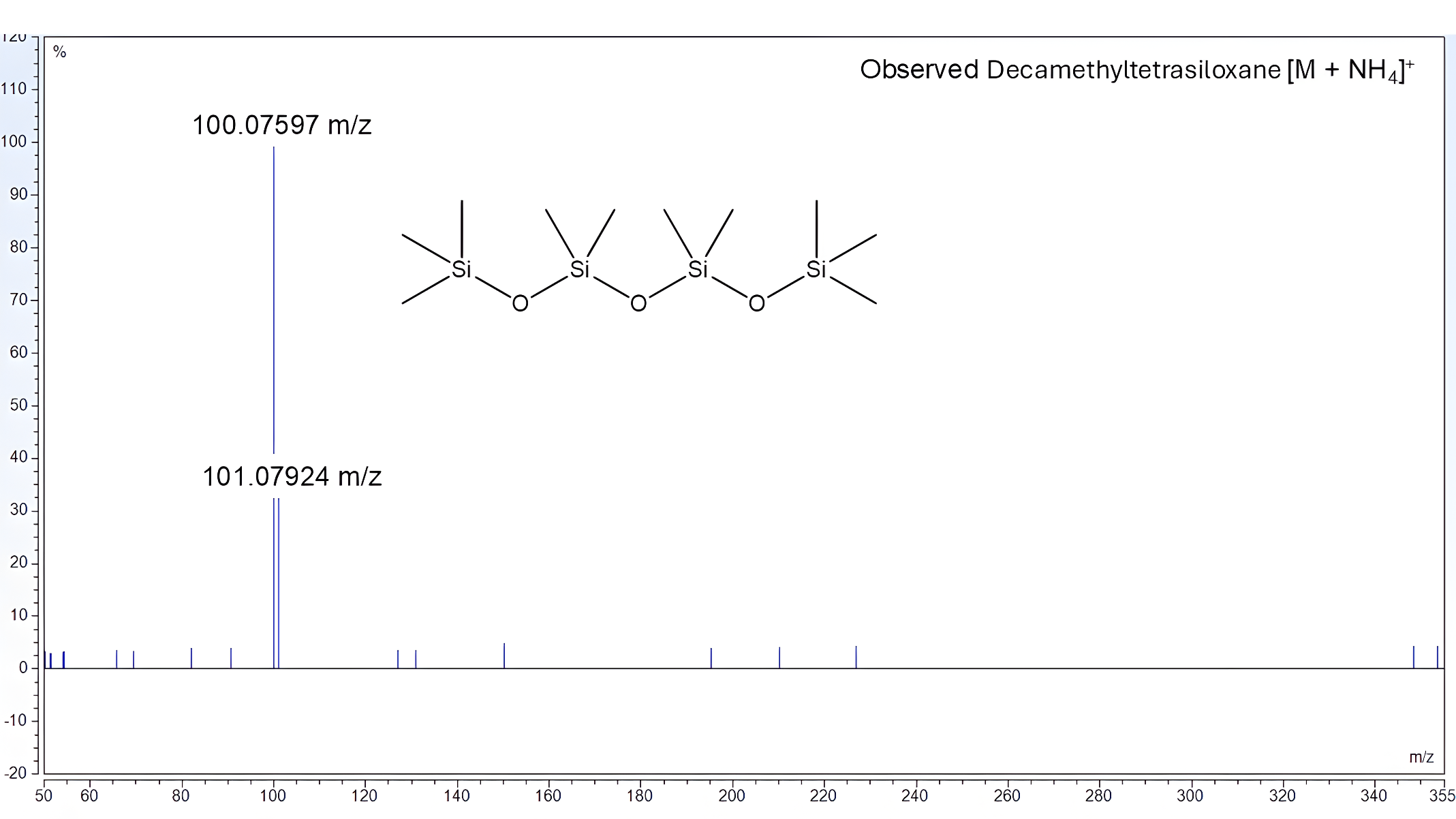
**

**Fig. S9.** Example of mass spectrum of Decamethyltetrasiloxane in BD Plastipak in positive mode**.**

**
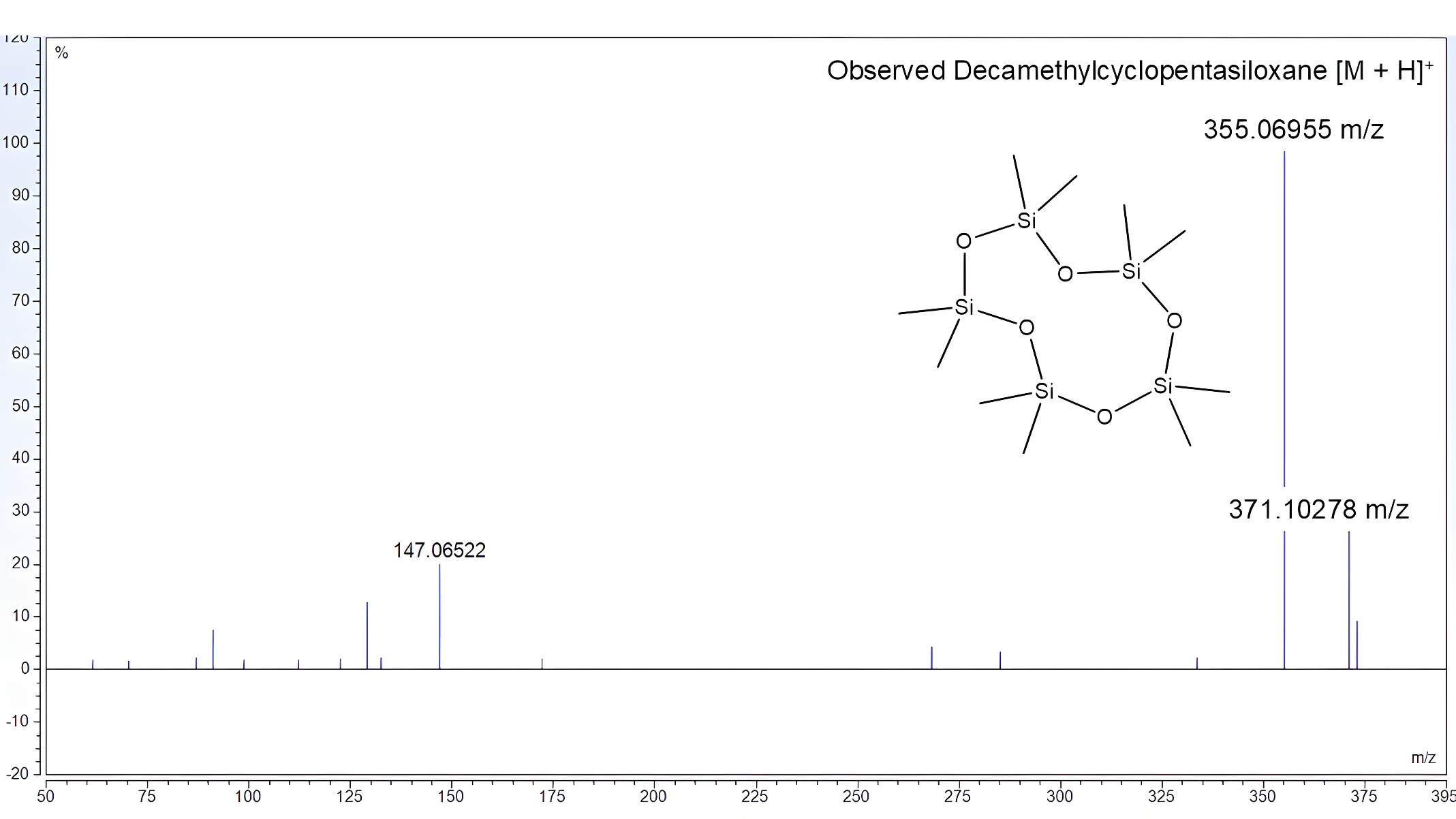
**

**Fig. S10.** Example of mass spectrum of Decamethylcyclopentasiloxane in BD Plastipak in positive mode**.**

**
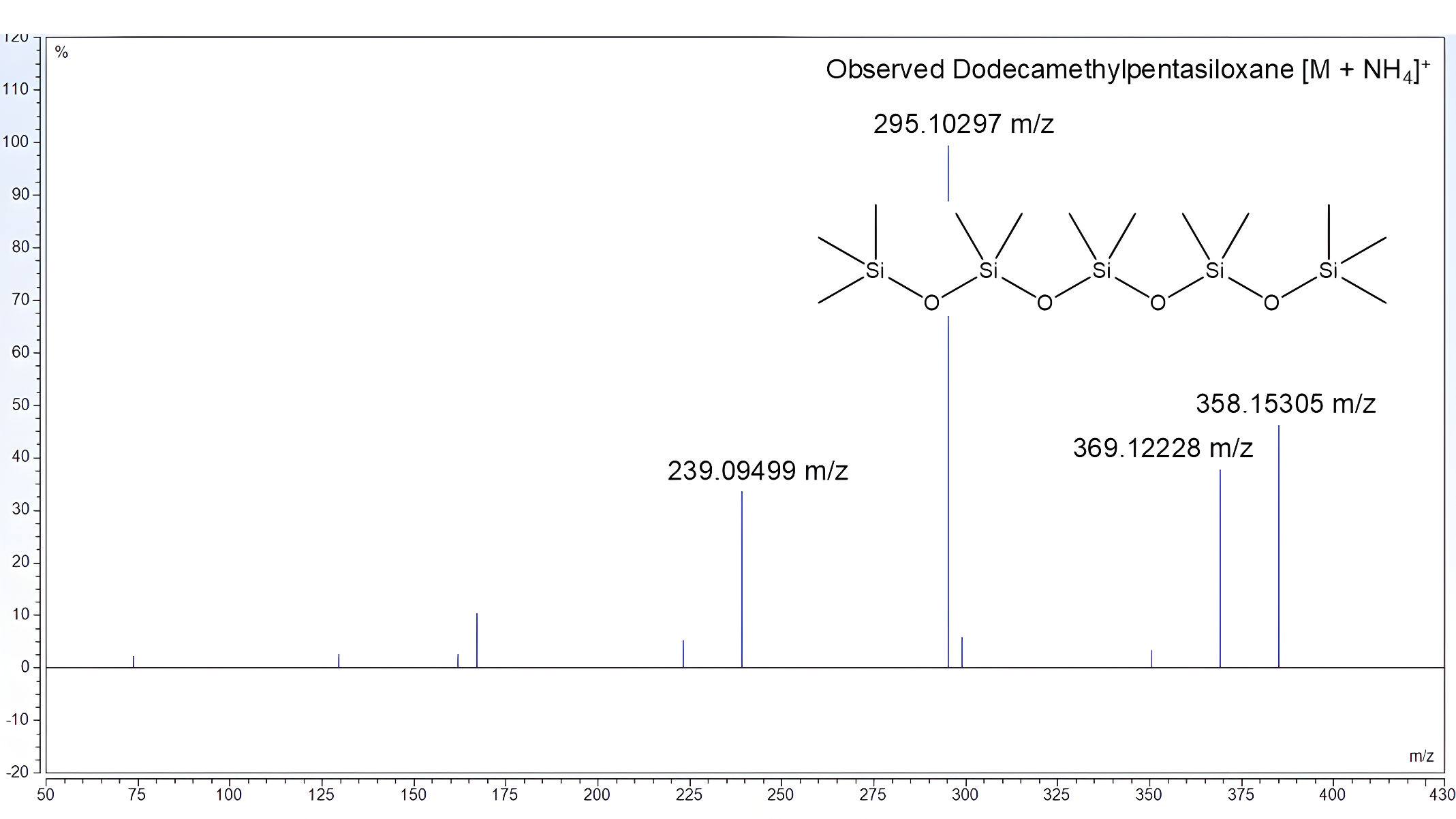
**

**Fig. S11.** Example of mass spectrum of Dodecamethylpentasiloxane in BD Plastipak in positive mode**.**

**
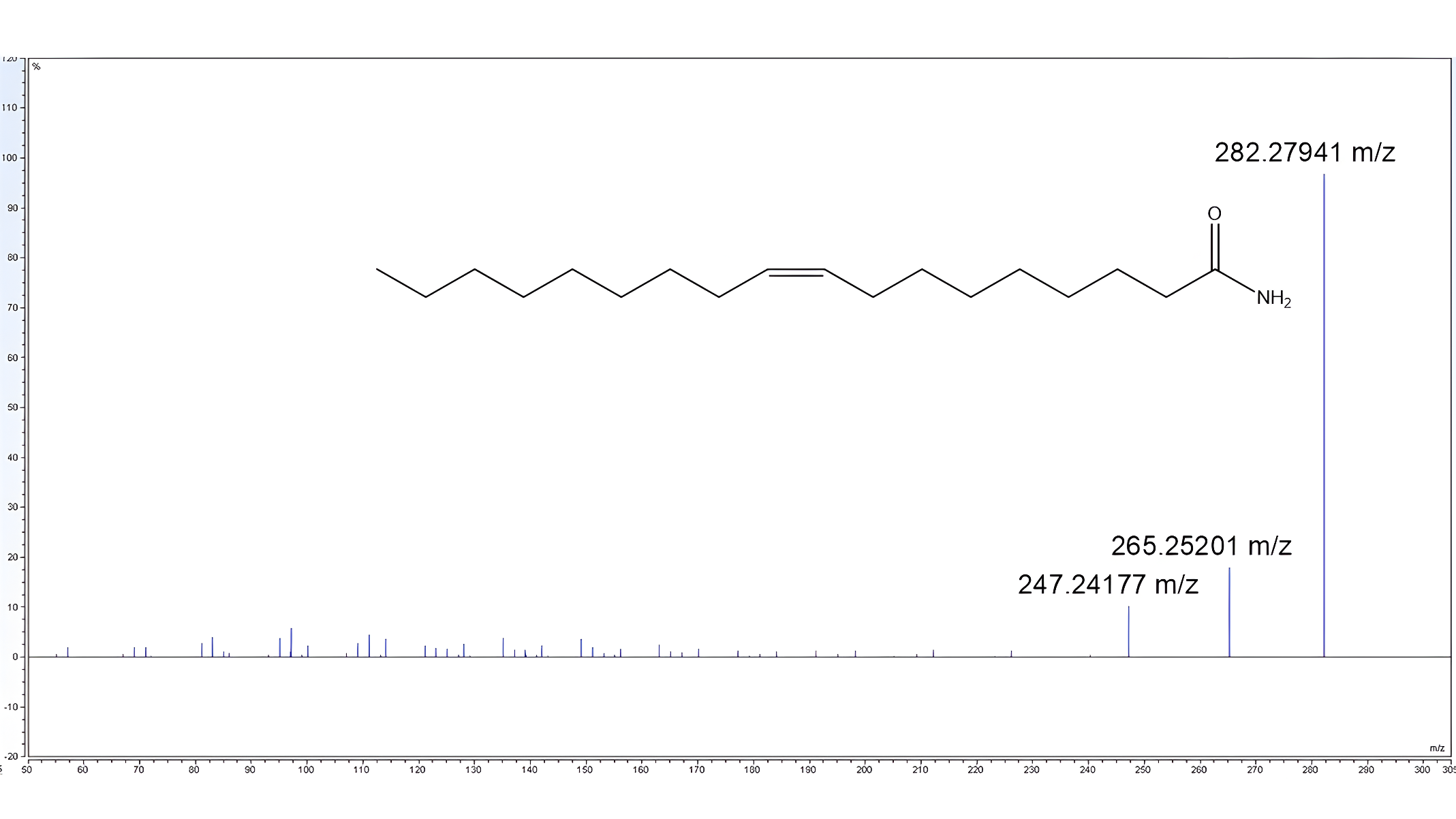
**

**Fig. S12.** Example of mass spectrum of Oleamide in BBraun Omnifix in positive mode**.**

**
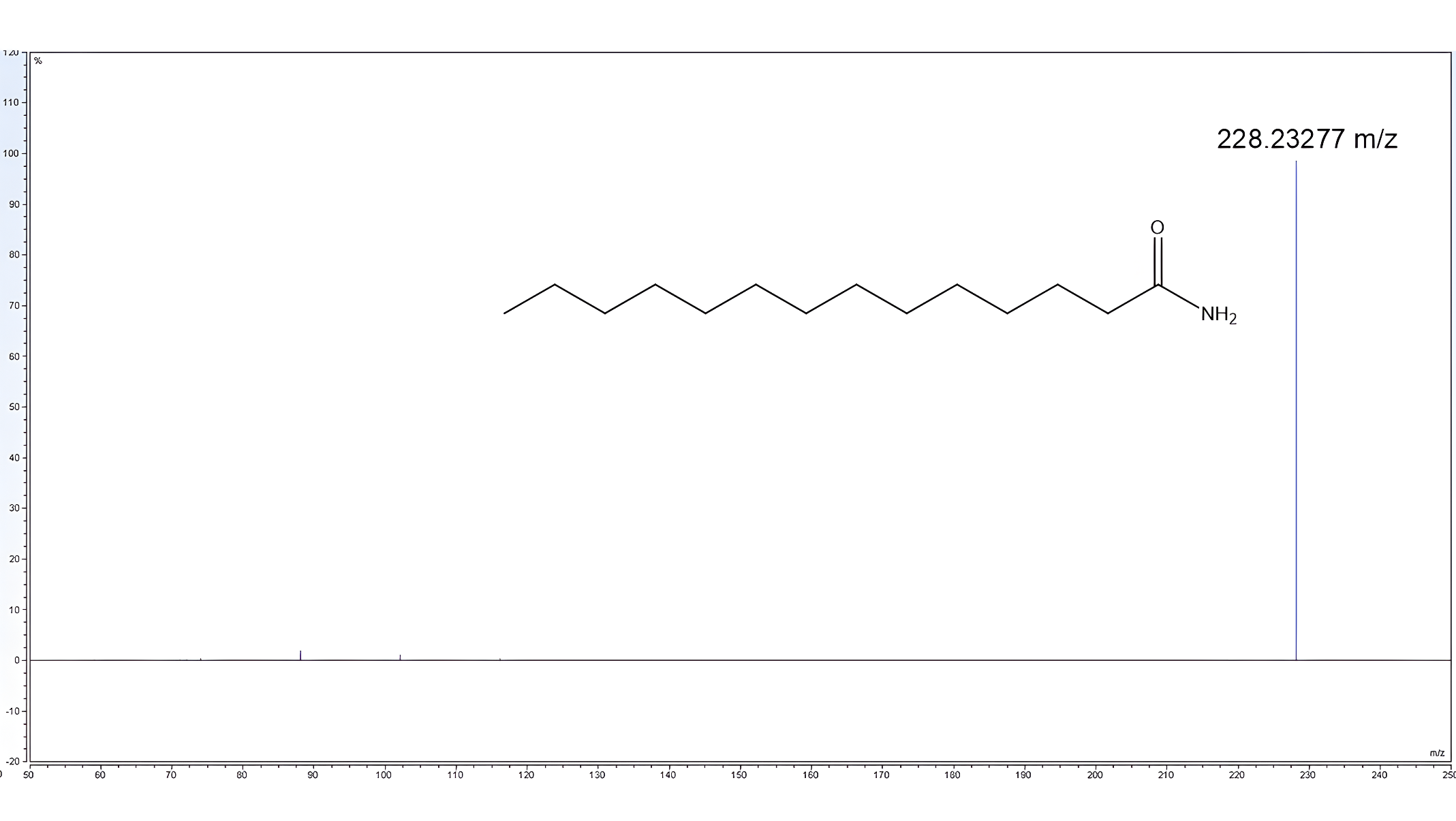
**

**Fig. S13.** Example of mass spectrum of Tetradecanamide in BBraun Omnifix in positive mode**.**
